# Extraction of Marine Bioactive Compounds from Seaweed: Coupling Environmental Concerns and High Yields

**DOI:** 10.3390/md23090366

**Published:** 2025-09-19

**Authors:** Carlos Cardoso, Joana Matos, Cláudia Afonso

**Affiliations:** 1 Department of Sea and Marine Resources, Portuguese Institute for the Sea and Atmosphere (IPMA, IP), Avenida Alfredo Magalhães Ramalho, 6, 1495-165 Algés, Portugal; joana.matos@ipma.pt (J.M.); cafonso@ipma.pt (C.A.); 2 CIIMAR (Centro Interdisciplinar de Investigação Marinha e Ambiental), Interdisciplinary Centre of Marine and Environmental Research, University of Porto, Rua dos Bragas 289, 4050-123 Porto, Portugal

**Keywords:** innovative extractive routes, ‘green’ solvents, biorefinery, wet route, selective extraction, seaweed biomass

## Abstract

This review examines recent advances in the extraction of valuable compounds from seaweed biomass, focusing on practical feasibility and environmental sustainability. There is a growing importance of seaweed biomass in terms of the study and acknowledgment of its untapped biotechnological potential (multiple compounds and biological activities) and in terms of economic impact. Conventional extraction techniques largely fail to address this challenge, even if optimized. This has led to the development and testing of innovative technologies as solutions for a ‘green’ and effective extraction of components from seaweed biomass and to biorefinery processes. There are large differences in outcomes between alternative processes, depending on the matrix, operational parameters, and targeted compounds and activities. Despite the positive results of some techniques, such as those based on physical mechanisms, namely Microwave-Assisted Extraction (MAE) and Ultrasound-Assisted Extraction (UAE), and on enzymatic selectivity, i.e., Enzyme-Assisted Extraction (EAE), there is no universally effective technique and approach, thus justifying integrated approaches combining different techniques. The application of ‘green’ solvents was also assessed and proven to harbor a large potential, just as the wet route. Although technical difficulties, outcome variability, and economic viability problems are relevant, recent progress in seaweed processing paves the way for a future blue economy.

## 1. Introduction

Among the wide variety of marine life forms, seaweeds are a group of photosynthetic organisms with an important role in the capture of carbon and oxygen production, thereby constituting a significant share of the marine biomass. There are three main seaweed groups, which differ genetically, biochemically, and in their overall phenotype: Chlorophyta (green algae), Rhodophyta (red algae), and Phaeophyta (brown algae) [1].

Research has shown that the photosynthetic efficiency of marine seaweeds is substantially higher than that of terrestrial plants [2,3]. On the other hand, their biomass is rich in a wide variety of nutrients and biologically active compounds [4]. In particular, their biomass contains valuable polysaccharides, lipids, and proteins as well as micronutrients, such as vitamins, minerals, or sterols, but also secondary metabolites, such as polyphenols and pigments [5,6]. Some species, such as *Enteromorpha intestinalis*, *Palmaria palmata*, and *Vertebrata lanosa*, have been pointed out as containing high-quality profiles of essential amino acids and lipids [7,8]. Moreover, biological activities, such as anticoagulant, antidiabetic, anti-inflammatory, antimicrobial, antioxidant, anti-tumoral, antiviral, hypocholesterolemic, immunomodulatory, and prebiotic, have been found in seaweed [4,9].

Extracting these biologically active compounds and attaining extracts with high activity is a major challenge [10]. The usual extraction processes, still much applied by the industry worldwide, may lead to relatively low yields and/or decomposition of some valuable substances as a result of excessive temperature and processing time, notwithstanding permanent optimization efforts, for instance, using Response Surface Methodology (RSM) and similar approaches [10]. Cell disruption and, in particular, destruction of the cell wall barrier, is a key factor, but also other physicochemical phenomena affect, modulate, and thwart the release of the bioactive components [11,12,13].

Usual methods for extracting bioactive compounds from seaweed biomass typically involve several steps, being affected by factors such as temperature, extraction time, solvent choice, liquid-to-solid ratio, and flow rate [14,15,16]. The most common extractive process, Solid–Liquid Extraction (SLE), involves the direct utilization of liquid solvents as extracting agents [16]. There is also percolation (PE), which is an extraction technique that is distinguished from SLE by its continuous mode of operation. A third usual technique is Reflux Extraction (RE), which is based on a continuous reflux (repeated solvent evaporation and condensation) [8,15].

However, traditional methods are quite time consuming and may have safety and toxicity issues associated depending on the extractive solvents, as well as negative environmental impacts at an industrial scale [17]. These environmental sustainability concerns have to be considered together with efficiency/yield issues in the development of a seaweed-based industry viable in the long-term [18,19].

In this context, alternative and so-called ‘green’ solvents and processing techniques [20,21] are emerging, undergoing research and development, and, in some instances, already maturing and being introduced into the seaweed processing industry. In many cases, these alternatives can consistently bring about higher process yields, shorter processing times, and ensure higher biological activity in the final extracts/fractions [19]. Such innovative extracting techniques correspond to a wide variety of processes of physical, chemical, and/or biological nature, comprising Microwave-Assisted Extraction (MAE), Ultrasound-Assisted Extraction (UAE), Pulsed Electric Field (PEF), Sub-critical Water Extraction (SWE), Pressurized Liquid Extraction (PLE), Supercritical Fluid Extraction (SFE), High Hydrostatic Pressure (HHP), Solid-Phase Extraction (SPE), Solid-Phase MicroExtraction (SPME), Enzyme-Assisted Extraction (EAE), Osmotic Lysis (OL), Alkali Extraction (AlE), pH-shift Extraction (pHE), and other technologies [8,16,19,22,23,24,25,26,27,28,29].

Beyond the application of novel extractive methodologies and operational conditions, there are also innovative approaches and transforming concepts. Among these, biorefinery is a key concept whose gist is the integral valorization of the whole seaweed biomass through a thorough knowledge of all its components and the clever alignment and articulation of extractive and separation processes in order to maximize each specific extractive yield and enlarge each output in the form of varied applications in the fields of feed, food, cosmetic, pharmaceutical or specialty biotechnology [25,30,31,32]. Seaweed is considered one of the most promising bio-feedstocks for renewable fuel production [33], requiring sunlight, water, nutrients, and carbon dioxide (CO_2_) to generate energy [3], but not demanding precious arable land. There have been some relevant recent studies [34] concerning biorefinery and its zero-waste approach to specific seaweed species, such as the green seaweed *Codium* sp., thereby showing their potential [34,35].

Another innovative approach with the purpose of environmental sustainability is the Wet Route (WR) extraction of targeted compounds, which has been proposed as a less energy-intensive and operative way to have a biorefinery approach in the case of algal biomass [29,36]. In this approach, cell disruption operations are performed before extraction, thus improving the release of valuable molecules from the disrupted algal biomass [36]. In addition, dewatering and drying operations—usually performed before extraction—are rendered unnecessary. This is important since these are energy-intensive procedures. The energy required for dewatering/drying represents more than 80% of the total energy consumption in the extractive process.

Nonetheless, it should be noted that most of these novel technologies, approaches, and concepts have neither stood the test of time nor met a large-scale implementation. In many instances, they cannot yet be performed at an industrial scale. This reality has also led to a substantial number of studies using Life Cycle Analysis (LCA) as a means to assess the sustainability of the proposed value chains [37]. In this regard, it has been pointed out that seaweed biorefineries have the ability to stimulate the growth of the blue economy and are a promising economic opportunity within the higher-value products market [37].

**Figure 1:**
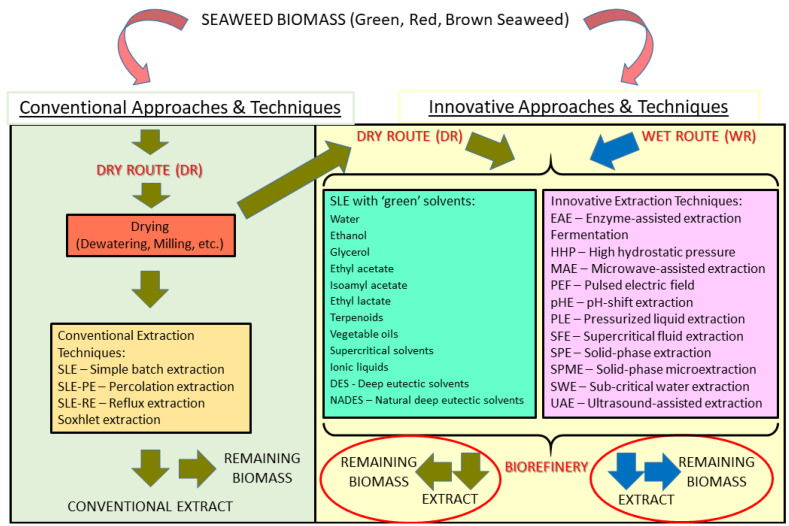
Overview diagram of possible alternative strategies and extraction techniques to be applied to seaweed biomass.

The objective of this review was to produce a thorough assessment of the state-of-the-art regarding not only extractive methods and processing approaches already applied to the seaweed biomass, but also emerging innovative techniques and new concepts/approaches that may represent an advancement to the current state of affairs, especially if taken under a new perspective of biorefinery and blue economy (Figure 1).

### Literature Search Procedure

A thorough literature search was planned, systematically carried out, and critically assessed at each step. The literature screening included books, scientific papers in peer-reviewed and indexed journals, official reports, communications in congresses and other conferences or seminars, as well as other publications considered scientifically significant. Important databases comprising the field of phycology and related fields were searched, namely, ScienceDirect, SpringerLink, PubMed, among several others. In addition, broad web searches (encompassing official bodies) were performed, but were circumscribed to publications (books, papers, etc.) written in English. Keywords used were seaweed, algal biomass, phycology, extraction, extractive techniques, extractive yield, conventional techniques, emerging technologies, purification, separation, refining, nutrients, bioactive compounds, biological activity, biorefinery, blue economy, sustainability, biotechnology, and high-value-added applications. Approximately 800 papers and documents were assessed. A short list of 305 most significant publications was attained after screening the abstracts.

## 2. Conventional Extractive Routes and Techniques

Seaweed biomass already has a long history of extraction aiming at specific components, such as carrageenan or agar [25,38]. However, as already stressed above, seaweeds also contain a variety of other components with invaluable nutritional, functional, and biological properties. This has led to the application of conventional extraction technologies—usually already applied to terrestrial plants and other matrices—to seaweed biomass. Extraction in its broader sense also encompasses processes for concentrating specific components. Indeed, for instance, secondary metabolites with biological activity may need to be concentrated; that is, they may have to be extracted from the seaweed biomass and, at least, partially isolated in a more purified fraction, which is then appropriate for various applications. All these aspects raise questions about how to choose effective, safe, reliable, and sustainable methods for seaweed extraction. It is critical that employed techniques do not cause loss of the compounds targeted by the extractive method (e.g., as a result of high temperature) or lead to the generation of dangerous compounds. Furthermore, it should be remarked that extraction processes able to be scaled up to an industrial-scale level may entail condition sets that are more drastic and, also, automatically exclude expensive technologies used for microextraction and/or analytical purposes [39]. Accordingly, there is a balance between sustainability, product quality, practicality, and technological and commercial viability that must always be carefully considered. In this context, the so-called conventional approaches and methods [16] offer the advantage of having already proved their worth in various circumstances and for many different matrices and compounds.

### 2.1. Main Conventional Routes

#### 2.1.1. The Conventional Approach

There is a traditional way of thinking and tackling the issue of extracting specific components from a biological matrix, and this has also been applied to seaweed biomass in several instances [16]. Indeed, seaweeds are usually subjected to a pre-treatment that involves drying and grinding prior to any extraction procedure. This corresponds to a DR approach, which is the most conventional route [16]. Though seaweed biomass can be sun-dried in favorable latitudes and places as well as propitious seasons that allow for this procedure, the most common drying technique involves the operation of ovens, which may be solar or, most often, electrical, and may involve only heating, or heating and forced air circulation, thereby entailing a high energy consumption and carbon footprint [16]. Another conventional procedure involves prior mechanical dewatering as a way to stabilize seaweed and reduce the cost of downstream transport and drying [40]. However, this also has drawbacks, since some water-soluble components may be lost instead of remaining deposited on the biomass after heat-driven drying. Regarding mechanical processes for reducing particle size and other purposes, grinding, milling, and extrusion may also be mentioned as relevant, and a previous mechanical treatment is always critical for the optimization of extraction, thereby setting the stage for more advanced and efficient seaweed processing techniques [41,42]. Indeed, these mechanical pre-treatments are usually carried out prior to chemical and biological processing, being employed with the purpose of reducing seaweed biomass to approximately 2 mm, resulting in powdered samples [41,43]. Furthermore, extrusion is another possible mechanical technology, which can be integrated into the seaweed processing flowsheet as a pre-treatment step when conversion of cellulosic material into biogas is an objective [42]. There are other pre-treatments that may be applied to seaweed biomass, but these concern specific extraction purposes. Namely, there are physicochemical pre-treatments for preparing biomass to enzymatic action [44,45]. Overall, these technologies that aim to prepare raw materials for downstream extraction have been previously developed for cultivated land plants and then applied to a wide variety of matrices, including seaweed.

The conventional approach to seaweed extraction and valorization of the biomass main fractions can be generalized to some extent, being divided into pre-treatment of the raw material, extraction in single or multiple stages (depending on the existence of one or various compounds with significant commercial value), separation of the valuable compounds from residues, and downstream recovery and concentration of the final products [19]. This conventional route also leads to large amounts of waste—wherein the remaining biomass after extraction of the valuable components is a major part—and by-products. Most of these materials are typically treated as waste, being frequently sent to landfills or incinerated, or given low-value-added applications, for instance, as an undifferentiated component of soil fertilizers [46]. This type of single-purpose extraction aiming at one component (or a few components) is still ubiquitous and it is quite deleterious for the environment and the sustainable use of marine resources.

#### 2.1.2. Conventional Thermal Pre-Treatments

There has been little progress in minimizing a wasteful approach, which may be explained by the difficulties and hurdles associated with introducing any major change into the conventional flow diagram. A first major hurdle lies in overcoming thermal pre-treatment. In fact, one of the main reasons for performing an energy-consuming process such as seaweed drying lies in the risks of degradation of the biomass due to a high level of water activity [47]. For instance, sugar kelp (*Saccharina latissima*), a brown seaweed species, is highly perishable as a result of its high moisture content (approximately 92%, *w*/*w*), thus being dried for industrial processing. For achieving this, the biomass is either conventionally sun-dried or dried with hot air [47]. However, the previous approach is quite slow, requires adequate weather conditions, and, unless special measures are taken, it may result in the exposure of seaweed biomass to pest or animal infestation [48]. On the other hand, the latter approach is energy-intensive while having been shown to be better than sun-drying regarding nutrient retention and hygiene [49]. Another important example concerns eucheumatoid (red) seaweeds that are a source of carrageenan and, after harvesting, need to be dried from a moisture content surpassing 90%, *w*/*w*, to the industry standard of less than 38% moisture [50]. The drying process is crucial since the reduction in water activity inhibits microbial growth, thereby preventing a deterioration of carrageenan quality [51]. Seaweed dried to an adequate moisture level can be kept for years without any loss of the gel-forming properties. Given this main advantage, various drying alternatives, ranging from solar dryers to powered ovens, have been employed in order to decrease drying time in an economically feasible and environmentally sustainable way, while protecting seaweed from environmental factors [50].

In this context, optimization efforts have been undertaken concerning drying and, especially, the technology of hot air drying [47]. Any optimization endeavor requires good knowledge and control of key operational parameters affecting drying, necessarily comprising temperature, moisture diffusion coefficient, difference in water vapor partial pressure between biomass and the environment, thickness, or surface area [52]. If air is circulated, the type and velocity of air circulation are also factors to be taken into account in any optimization effort. Though more energy-expensive, since hot dry air is usually circulated by a fan or blower into a chamber in order to guarantee a uniform heat distribution, this kind of drying technique provides for a higher rate of drying [53]. Using such a technique, samples of the green seaweed *Ulva rigida* were dried at up to 150 °C, and final moisture levels of nearly 10% were attained by Thunyawanichnondh et al. (2020) [54]. In comparison to solar drying, it has been reported that higher recovery of carotenoids and chlorophyll is achievable [55]. Therefore, oven drying has been selected as the most suitable solution for drying pre-treatment of seaweed biomass, for instance, with the purpose of extracting vitamin B12 [56]. Furthermore, the oven-dried brown seaweed *Durvillaea antarctica* was found to have a higher retention of phytochemicals than that prepared with solar, vacuum, freeze, and infrared drying [57]. However, higher drying temperatures and lengthier processing times negatively affected chlorophyll and phenolic compounds in *D. antarctica* [57]. Another relatively recent study [58] showed that brown seaweed species (*Ascophyllum nodosum*, *Fucus vesiculosus*, and *Fucus distichus*), after being subjected to air drying, could be stored for up to a year without any loss of important nutrients and biologically active compounds.

Optimized drying procedures are thus essential to reduce costs, minimize waste, and enhance the profitability of seaweed drying techniques. Combining different drying techniques can also be a solution by making the best of the merits of each individual technology and thus helping to achieve efficient and sustainable drying processes [53]. Each drying method has advantages and disadvantages, being critical to evaluate the optimal method for specific seaweed types and to take into account the intended product quality, processing costs, and targeted market [53].

#### 2.1.3. Conventional Mechanical and Other Pre-Treatments

Regarding mechanical extraction processes, such as grinding and milling, these are pre-treatment solutions that are more environmentally friendly and safe—in terms of safety to human consumers—than the thermal techniques [59,60]. This has to do with their general absence of waste generation. Bead Milling (BeM), Mammer Milling (HaM), Ball Milling (BaM), and High-Pressure Cell Disruption (HPCD) are paradigmatic cases of such mechanical methods [59,60,61]. However, there may be practical hurdles in the employment of these methods. Namely, Teo and Wahab (2020) [60] have reported that the application of HPCD to seaweed biomass can lead to clogging problems, and BaM has been deemed unsuitable due to its relatively low rotating speed and downstream low extraction yields. Hence, BeM has been argued to be a better solution—for instance, it has been pointed out that it causes a rapid cell disintegration through this mechanical procedure, with the additional advantage of being an energy-saving process [62]. In addition, BeM does not raise safety issues, being a contamination-free process that does not generate any waste stream [59]. It is also possible to scale up BeM to an industrial-scale production and further increase extraction yields [63]. The technique has also been tested in *Kappaphycopsis cottonii* and concluded that its total operating cost was lower than alternative methods [59].

Mechanical pre-treatments require further study, and their optimal operational parameters must be found. Namely, a study by Vanegas et al. (2014) [64] that targeted the extraction of reducing sugars, lipids, and proteins from two relevant seaweed species in Ireland (*Laminaria digitata* and *Saccharina latissima*), analyzed several thermal, mechanical, and chemical pre-treatments and their conditions. This study observed that each pre-treatment enhanced the release of macromolecules to a different extent when compared to non-treated control batches. The authors concluded that, among the pre-treatments studied, a combination of milling without beads and under cryogenic (liquid nitrogen) conditions, and a chemo-thermal procedure ensured the strongest effect in releasing sugars and lipids from both seaweed species [64]. Precisely, such findings highlight the relevance of seaweed pre-treatment and the need to perform optimization studies. This was also performed in a study that aimed to optimize a mechanical pre-treatment to destroy the seaweed’s physical barriers to extraction [42]. In particular, a Hollander beater mechanical pre-treatment was applied to a batch of Laminariaceae and assessed by Tedesco et al. (2014) [42]. The derived biogas yield was used as a response in order to identify the optimal input variables in a specific RSM design [42]. In this case, the best results were achieved after 10 min using the minimum gap (76 mm) in the Hollander beater and incubation at 50 °C. Hjorth et al. (2011) [65] tested extrusion pre-treatment in different biomass materials with the purpose of increasing biogas production further downstream. It was observed that extrusion accelerated the degradation of slowly degradable organic compounds, being the methane yield significantly increased, up to 70% after 28 days [65]. More such studies on different pre-treatments with potential practical industrial applications are needed.

### 2.2. Conventional Extractive Techniques

#### 2.2.1. General Overview

The conventional routes of processing seaweed, which are outlined above, are usually associated with typical extractive techniques. These are mostly variations on a solid–liquid interaction and separation process, encompassing SLE, PE, and RE [8,16]. Though the procedure may vary, ranging from simple batch mixture to percolation and reflux, solvents, such as water (and aqueous solutions), ethanol, methanol, ethyl acetate, chloroform, and acetone at room or higher temperature, are common to these processes. The choice of a particular process depends on factors such as the chemical nature of the targeted biologically active compounds, the specific characteristics of the algal matrix, intended yields for achieving commercial viability, and the type of application—whenever there is a final human consumer, standards are higher, and safety issues are of paramount importance [22]. Regarding the solid–liquid interaction with utilization of seaweed biomass as starting material, it should be remarked that many solutes are found inside algal cells, while others are part of the cell walls (usually in polymerized form), thereby also affecting extracting technique choice [66]. As a consequence, the degradation of cell walls, which act as a barrier to component release, enhances mass transfer. Usual stages in the solid–liquid interaction are as follows: (i) solvent diffusion within the seaweed matrix; (ii) hydrolysis and solubilization of targeted component(s); (iii) diffusion of the component(s) through the seaweed matrix; and (iv) mass transfer to the bulk solution [66]. It has often been reported that the third stage is the rate-limiting step and that a smaller particle size and effective cell wall degradation are essential for a successful extraction [66]. Any solid–liquid extraction process is limited by the capability of sorbent sorption of the targeted component(s) and requires a small particle solid-phase separation, which conventionally is performed by centrifugation or filtration [67,68]. Indeed, in the conventional techniques, key operational parameters typically involve the type of solvent, particle size of the solid (in general, powdered seaweed biomass), time and temperature, number of extractive cycles, or stirring velocity. All these can significantly impact extraction efficiency as well as the quality of the attained biologically active substances [69]. Therefore, selecting suitable extractive technology and having fine-tuned operational parameters is fundamental for achieving the intended outcome [22].

More specifically, for extracting phenolic compounds, polar solvents with low boiling points, such as ethanol, methanol, acetone, or a mixture of acetone and water, are generally considered the standard choice [68]. In addition, for water-soluble amino acids, peptides, simple carbohydrates, and nucleotides, water or aqueous extractions are advised [70]. On the other hand, for moderate hydrophobic products, with low or intermediate polarity, an ethyl acetate extraction is frequently advocated [71]. In the case of lipophilic and totally hydrophobic substances—for instance, sterols and other lipid groups—, acetone, chloroform, or, alternatively, chloroform in combination with methanol are conventional choices [72,73]. Indeed, in the specific case of lipid extraction, the Folch [74] and Bligh and Dyer [75] techniques have been traditionally employed, taking advantage of polarity differences between chloroform, methanol, and water to form an extraction system comprising both an SLE phase and a liquid–liquid partition phase. More recent experimental work [76] has shown that the substitution of dichloromethane (CH_2_Cl_2_) for chloroform (CHCl_3_) kept acceptable yields—similar to the older methods—while mitigating detrimental health and environmental effects. For carotenoids, a lipophilic group of substances, ethanol extraction at 100 °C has been shown to be effective in extracting, for instance, fucoxanthin from the brown seaweed *Himanthalia elongata* [77]. An overview is provided in Table 1.

#### 2.2.2. Specific Case of Seaweed Polysaccharides

For polysaccharides, a key component of seaweed biomass, given the practical complications of extracting polysaccharides by a single technique, a combination of extraction and purification techniques has usually been adopted as the best strategy [95]. The chemical composition and biological activity of polysaccharides necessarily play a major role in selecting the most adequate extraction procedure [96]. Aqueous extractions, comprising diluted acidic extraction, as well as other chemical-based techniques, have been employed and are considered the conventional first approach of extracting seaweed polysaccharides [95,97]. These techniques are generally inexpensive, compatible for functional food preparation, and friendly to the environment, but their efficiency is considered to be too low [95,98]. Additionally, it should be noted that hot solvent and extended periods of extraction are often necessary in such conventional methodologies, leading to the degradation of natural biologically active compounds [99]. Rioux et al. (2007) [100] proposed a stepwise process beginning with crude extraction using ethanol, t-butanol, petroleum ether, and chloroform solvents—to separate proteins, pigments, and other components—and followed by residue treatment with diluted acid/water, thermal hydrolysis, and, if necessary, calcium chloride-induced precipitation of alginic acid (and other polysaccharides). Isolation of polysaccharides is usually achieved with polar solvent precipitation, such as ethanol and acetone, in which polysaccharides are insoluble, followed by polysaccharide separation with downstream techniques like membrane filtration or centrifugation [100].

The extraction of fucoidan from brown seaweed offers a paradigmatic example of a conventional process applied to seaweed biomass [66]. The biomass is firstly subjected to pre-treatment (washing, milling, and drying), then to filtration/centrifugation resulting in a defatted intermediate product, to an extraction that may use water, acid, alkali or ethanol, again to filtration/centrifugation, and, finally, to a purification step—through either ethanol/acetone precipitation, liquid–liquid fractionation, ion-exchange chromatography, size-exclusion chromatography, affinity chromatography or membrane filtration [66]. There are plenty of studies [66] on variations in the conventional extraction of fucoidan, ranging from aqueous extraction applied to dried *Ecklonia cava* at 70 °C [101], to alkaline extraction from *Sargassum stenophyllum* with 4 M potassium hydroxide (KOH) at room temperature [102] and to acid extraction from *Saccharina japonica* with 0.05 M hydrochloric acid (HCl) at 25 °C [103].

This panorama can also be found in the extraction of other seaweed carbohydrate components, such as carrageenan in red seaweed [104] or ulvan in green seaweed [105]. For carrageenan, after a prior drying step to prevent degradation, subsequent steps comprise washing to remove impurities, a hot alkali extraction process to release carrageenan from cells, clarification, and reduction to powder [104,106]. Further downstream, different techniques are used to isolate carrageenan from solution, either freeze–thawing—also used for agar [107]—or alcohol precipitation or potassium chloride precipitation. In freeze–thawing, carrageenan-containing solution is subjected to gelling with different salts, resulting in freezing [104]. Afterwards, thawing enables water separation, resulting in a product composed of carrageenan and its salt and requiring grinding to attain the intended particle size. If the alcohol precipitation method is used, the carrageenan solution is subjected to precipitation by 2-propanol or other alcohols, followed by alcohol evaporation, drying, and grinding to the targeted particle size [104]. Potassium chloride precipitation encompasses volume reduction through evaporation, extrusion, washing of the attained gel threads, pressing, drying, and milling to obtain carrageenan powder [104]. With respect to ulvan, dried seaweed biomass undergoes a solid–liquid interaction treatment, a kind of RE technique (utilization of Soxhlet extractors), followed by static batch extraction with water, filtration, concentration, deproteinization, and, finally, precipitation and freeze-drying of the polymer [105].

#### 2.2.3. Utilization of Conventional Extraction Techniques at an Industrial Scale

The seaweed industry is still largely based on these conventional extractive technologies, especially in the case of algal hydrocolloid polysaccharides, such as agar, alginate, and carrageenan, traditionally used by Western countries as stabilizing, thickening, and gelling agents in the food industry [108]. Meanwhile, France has authorized the utilization of seaweed for human consumption as vegetables/condiments, thus creating new opportunities for the industry [109]. Currently, the seaweed processing industry is a multi-billion-dollar business, of which 85% corresponds to food products for human consumption, representing agar, alginate, and carrageenan almost 40% of the global hydrocolloids market [108]. However, it should be noted that less than 1% of total seaweed production is directly used as food, hydrocolloids being the major final products [108].

Conventional industrial production of these hydrocolloids is still dominant and involves large equipment, such as huge mixing vessels and potent filtration units, for performing multi-stage processes, with washing, pre-treatment, solid–liquid interaction, precipitation and filtration, final drying and milling as main steps [107,110]. Hot water is the most usual industrial solvent for extracting hydrocolloids from seaweed biomass, since these polysaccharides are water-soluble with the noteworthy exception of alginate/alginic acid, which demands a hot alkali solution as an extracting agent [110,111]. Indeed, alginate in the seaweed biomass is found in the form of water-insoluble salts, requiring the presence of an alkali medium to convert them into water-soluble alginate salts. Alkali treatment is also applied by the industry in the agar and carrageenan processes because hot water extraction leads to hydrocolloids with a lower gel strength [110]. The alkali treatment can be performed before or during extraction and converts the 6-sulfated macromolecules into 3,6-anhydrogalactose polymers with a concomitant improvement of the gelling properties [110]. Furthermore, a very dilute-acid treatment is sometimes coupled with heat to improve water penetration, for instance, in the case of the industrial process of agar extraction from the red seaweed belonging to the genus *Gelidium* [107]. Industry favors the alkali procedure, despite its lower yields—a consequence of polysaccharide degradation under alkali conditions and inability of alcohol addition to induce precipitation of the formed low molecular weight polymers—, given the commercial demand for high gel strength hydrocolloids [106,112,113,114]. In spite of this and other problems, these chemically driven processes are still most commonly used in factories that process seaweed worldwide [110].

#### 2.2.4. Optimization of Conventional Extraction Techniques

All the above-described conventional techniques have important drawbacks, flaws, and limitations that have thwarted the development of the seaweed processing sector and, as based on older technologies and approaches, are unable to meet the current high standards in product quality, safety to the end consumer, and environmental protection. In fact, conventional techniques have failed to achieve high recovery rates of several biologically active compounds from seaweed biomass, including lipophilic substances, specific carbohydrates, or terpenoids [115]. This difficulty is related to the rigidity of the seaweed matrix, which hinders the release of many substances, and remains an unresolved problem in seaweed processing with conventional technologies [116]. The chemical composition of the seaweed matrix also has a major influence on the disruption efficiency and yield in targeted compounds [117]. Moreover, these older techniques are very often time consuming, may require large quantities of polluting solvents (as described above), which can result in environmental problems and safety issues with the presence of contaminants in the end products, losses due to volatilization during concentration stages, large quantities of waste and undervalued by-products, loss of functionality of sensitive molecules, and lower purity levels [14,19,21,72,118,119,120]. These reasons have led to a search for alternative techniques with higher efficiency/selectivity or to efforts toward an upgraded and optimized utilization of conventional technologies.

Indeed, given the shortcomings and flaws of conventional techniques, in order to maximize yield in the targeted component(s), it is of critical importance to optimize operational parameters (Table 1), in particular, to have optimal conditions for the applied solvent and the solid–liquid interaction [99,121,122]. In particular, within solid–liquid processes, it has been considered that RE, such as the Soxhlet extraction, may represent a progress with respect to simple maceration, since it consumes less solvent, while enabling higher purity and greater yields [14,123]. Though the reduction in utilized solvent quantities and generated waste also represents an advance in favor of the environment (with respect to SLE and PE), RE is still a technique that may use solvents harmful to the environment and human health.

In any case, even the simplest seaweed biomass SLE maceration with heat under agitation can undergo meaningful improvement [124]. These authors studied polyphenol extraction from the brown seaweed *Sargassum fluitans* and its optimization. For this purpose, they performed a complete factorial design 3^2^ with replicates in the center of the plane, being extraction time and ethanol concentration as independent variables [124]. While higher ethanol concentration had a deleterious effect on yield, lengthier extraction time did not matter so much. Based on coefficients provided by the mathematical model, optimal conditions for polyphenol extraction were established [124]. Under such conditions, 8.66 mg of total polyphenols was obtained per g of dry seaweed.

Moreover, the choice of extracting solvent is a critical parameter of almost all conventional techniques, being dependent on the targeted component(s)—whose solubilization must be maximized—, desirable selectivity, efficiency, and velocity of mass transfer as well as other processual aspects [125,126]. The heating component of conventional extractions is also crucial for a successful outcome. It requires a thorough analysis not only of the temperature–time binomial, but also of the type of heat transfer—considering the usual combination in conventional processes of convection through the solvent and conduction from the solid surface to the core of the biomass particles [72,125]. In fact, all the various operational parameters deserve thorough analysis and are optimizable. However, this multiplies the number of trials and the overall research investment that is needed to deliver meaningful advances, thus justifying the application of RSM and similar methodologies. Indeed, RSM and RSM-equivalents have been used to optimize conventional processes [10,42]. Namely, extraction conditions for *Gracilaria gracilis* were optimized with the help of such an experimental design technique for achieving high biological activity in the extracts [10]. Furthermore, the mechanical pre-treatment of *Laminariaceae* spp. biomass was also subjected to an RSM-supported optimization [42].

Even after optimization, the processes currently used present serious problems with high consumption of solvents, energy, and time as well as potentially damaging effects on nutrients and biologically active compounds whenever high temperature and lengthy extraction time conditions are required [125]. On the other hand, optimization itself is difficult because of the large variability of seaweed biomass as a raw material. This is particularly the case when initial contents and extraction outcomes are highly dependent on geographical source, harvesting season, maturity/life cycle phase, pre-treatment applied, storage history, particle size, among other not fully controlled factors [110,125]. Accordingly, due to common weaknesses of conventional techniques and approaches, other methodologies and strategies have been examined, including so-called ‘green’ or ‘alternative’ technologies, which may conjugate environmental sustainability and commercial viability [19,125].

## 3. Innovative Extractive Routes and Techniques

The problems and insufficiencies associated with the conventional approaches and technologies used for extracting nutrients and valuable components from the seaweed biomass have been a limiting factor in the full valorization of this marine resource, thus restricting the development of the industry and keeping a huge gap between the potential ascribed to seaweed and the current reality. For this reason, novel technological solutions and innovative strategies with upscaling possibilities and commercial viability are required. Precisely, commercial operation needs high recovery of the targeted components on the one hand, but concomitantly also the preservation of biological activity on the other, a combination that conventional techniques and approaches have failed to achieve [19]. In order to overcome all these limitations and also ensure a more environmentally friendly processing of seaweed biomass, alternative greener procedures and strategies have been developed, as presented in the sections below.

### 3.1. Biorefinery, Wet Route, and Other Innovative Approaches

#### 3.1.1. The Current Challenge

Firstly, the whole approach to the extraction of components from seaweed biomass must be rethought, and a full utilization of the biomass should be a key objective, not only from an environmental point of view (sustainability), but also in order to add more processual efficiency and economic value to this precious marine resource. These objectives demand processes with superior ability in overcoming the algal cell wall as well as other hurdles associated with the peculiar features of seaweed biomass [36]. Indeed, effective cell disruption methods are critically important for a high extraction yield of intracellular compounds, and it should be remarked that commercial extraction from seaweed biomass has been hampered by a lack of a comprehensive understanding of the cell disruption methods. High extractive yields are essential for an adequate separation of the components present in the biomass, regardless of being hydrocolloids, other polysaccharides, lipids, lipophilic substances, proteins and peptides, polyphenols, or other secondary metabolites. This separation, in turn, is indispensable for a rational and full utilization of all the biomass components to the utmost of their inherent value, that is, a biorefinery strategy. This is related to the aim of a zero-waste approach to seaweed biomass and the pursuit of a blue circular economy [34]. Moreover, besides reducing waste generation and other environmental impacts, a smaller carbon footprint with a lower level of energy consumption in the extraction process is a key goal. This may be reached with a rethinking of the whole extractive approach, namely through the avoidance of energy-intensive technologies, in particular thermal drying or hot extraction. The application of innovative cell disruption methodologies to wet biomass may also represent a gain in terms of yield and enable the implementation of a biorefinery approach—this may be called the WR approach, in contrast to the conventional DR. This new way of processing seaweed biomass requires not only novel technologies, but also a new way of thinking and a different underlying philosophy. Moreover, this is part of a larger whole, where economic activities and value are created from the sustainable and smart use of aquatic resources, which is the definition of blue bioeconomy by the European Commission [127].

#### 3.1.2. The Biorefinery Approach

The biorefinery approach is of critical importance for rethinking the future of seaweed valorization. It presupposes the integral valorization of the whole seaweed biomass through a thorough knowledge of all its components and the smart articulation of extraction/separation processes, thus maximizing each specific extractive yield and creating novel value-added products [25,30,128]. Additionally, the biorefinery approach is central to the bioeconomy concept, with seaweed presenting a huge potential to be used as a feedstock for it [129]. A recent systematic review has shown an overview of possible seaweed-based biorefinery chains and accompanying technical and non-technical difficulties to overcome for biorefinery to be successful in the realm of seaweed [130]. Full utilization of the seaweed biomass and minimization of environmental impacts are two sides of the same coin. In particular, seaweed biorefinery can substantially contribute to sustainable development by adding value to the original feedstock [32]. This involves transforming all seaweed biomass into a broad spectrum of applications/products using cascade conversion that may be adjusted to specific conditions.

In comparison to the situation of terrestrial biomass, the application of the biorefinery approach to seaweed biomass is less advanced, thus warranting further study [30]. As in many cases of terrestrial biomass, after extracting a valuable nutrient or biologically active component, there remains a large quantity of undervalued material. Just as with many instances of plant biomass, the conversion into biofuel and biogas may be an option and provide a route to a full biorefinery. Indeed, bioethanol production is more advantageous than aiming at lignocellulosics since it requires only hydrolysis and fermentation [30]. In this regard, it must be noted that, in general, seaweed fermentation requires specific microorganism strains with higher galactose metabolism ability than those used for terrestrial plants [131]. However, the crux of the matter is the rather high salt concentrations in seaweed material, leading to fermentation inhibition [30,132]. In any case, there are already some practical successful examples of attaining several commercially relevant components from seaweed biomass—encompassing case-studies of red, green, and brown seaweed species—, which may represent a meaningful step towards a biorefinery approach [29,30,34].

##### Biorefinery in Red Seaweed Species

In particular, red seaweed species have been considered an excellent raw material for this approach due to their valuable components, such as polysaccharides (including valuable hydrocolloids) and protein [25,30]. This biomass has the highest protein content among seaweeds, up to 45% w/dw, and its digestibility is higher than in other seaweeds [25,30]. Red seaweeds have also developed photo-protective defense mechanisms against UV radiation with the synthesis of pigmented compounds, such as carotenoids and phycobiliproteins, and mycosporine-like amino acids [133]. In the case of red seaweed processed for hydrocolloid extraction, it is common to only use 15–30% of the total dry biomass, leaving 70–85% as by-product or even waste [134]. For accomplishing a biorefinery approach, this material should be further processed to yield commercial products, taking advantage of its disrupted state for microbiological conversion or used to extract its protein—rich in essential amino acids—for multiple nutraceutical or food technological purposes [30]. Two case-studies on red seaweed species, *Gracilaria corticata* (agarophyte) and *Kappaphycus alvarezii* (carragenophyte), illustrate the potential and viability of biorefinery in this seaweed group [30].

For *G. corticata*, the spent biomass can be used to produce ethanol [135], thereby requiring a previous hydrolysis, hydrolysate separation by filtration after pH adjustment to 5.3, and nutrient supplementation for *Saccharomyces cerevisiae* fermentation. However, this fermentation would take 5 days at 30–34 °C, leading to an ethanol yield of 0.02 g/g [135]. Baghel et al. (2015; 2016) [136,137] went further and managed to design and propose one of the most thorough biorefinery strategies for red seaweed. The cascading process enabled a thorough utilization of the biomass, solvent recycling, absence of solid waste, and recovery of six different products, with a market value four times higher than the costs [137]. In detail, after pigment extraction, the remaining biomass was dried and lipids were extracted with a chloroform–methanol mixture—recovered and reused—, leaving a dried solid material whose agar contents were extracted with water at 120 °C for 1.5 h. Then, the spent biomass was hydrolyzed with a commercial cellulase to yield 0.27 g reducing sugar/g material and fermented with *S. cerevisiae* at 28 °C for 12 h [137]. This fermentation ensured a conversion into ethanol of approximately 90%, leaving a residue representing 12% of the starting material and usable as soil conditioner in accordance with its favorable C:H:N:S ratio [137]. In a global mass balance, for a ton of fresh biomass, that is 122 kg dry biomass, approximately 1 kg of pigments (R-phycoerythrin and R-phycocyanin), 2 kg lipids, 28 kg of agar, 15 kg of soil conditioner, and 4 kg ethanol would be attainable [137]. Hence, in dry matter terms, this biorefinery strategy would find, at least, a useful application for 40% of the biomass.

In the case of *K. alvarezii*, different biorefinery strategies have been proposed [138,139]. A WR strategy to support a biorefinery of *K. alvarezii* has been developed and aimed to extract an agricultural bio-stimulant and a semi-refined form of carrageenan [139]. Basically, fresh seaweed was washed and a sap was removed by filtration, leaving the solid material to be subjected to an alkali treatment with 8%, *w*/*w*, KOH solution in a proportion 1:1, *v*/*w*, at 70–80 °C for 1.5–3.0 h, and then filtrated and washed for the attainment of carrageenan [139]. The total process yields of the agricultural bio-stimulant and carrageenan were in the ranges 2.0–2.4% and 2.4–4.2%, respectively. This is still very insufficient, and the whole process is only a step away from a single-purpose conventional use of the biomass. On the other hand, it was possible to develop a *K. alvarezii* biorefinery with four end products: fertilizer, carrageenan, ethanol, and biogas [138]. This process started with a homogenization step coupled to a filtration for attaining a sap to be used as fertilizer, followed by drying, carrageenan extraction, two-step fermentation—a first step with *S. cerevisiae* for treating the spent biomass and a second step with *Escherichia coli* to convert the fermentation leftovers and the produced *S. cerevisiae* biomass—at 28 °C for 12 h, filtration/distillation to obtain ethanol, and final anaerobic digestion to yield the biogas [138]. The mass balance showed a ton of fresh seaweed, 670 kg of sap, and 73 kg dry biomass, from which 7 kg of carrageenan, 6 kg of ethanol, 6 kg of released CO_2_, and 7800 L of methane were measured. This represents progress with respect to previous research [139].

##### Biorefinery in Green Seaweed Species

Representative case-studies on green seaweed species’ biorefineries can be found especially for the genera *Codium* and *Ulva* [34,126,140,141,142].

For *Codium* sp., at the beginning of a recently proposed process [34], there was a hydroethanolic extraction, which enabled the separation of biologically active compounds (anti-inflammatory, anti-enzymatic, and photo-protective), and the remaining biomass was subjected to different carbonization processes, leading to a so-called biochar, that is, to a kind of biofuel. Since this remaining biomass had only been subjected to a single extraction, this whole approach seems to be unsatisfactory in terms of maximizing the value of the biomass components. For *Ulva* sp., a processing strategy was tested that was much nearer to a true biorefinery concept, thereby comparing a classical thermochemical hydrolysis followed by an enzymatic hydrolysis with a SWE hydrolysis [142]. In a nutshell, this biorefinery comprised drying and grinding *Ulva* sp., then the hydrolysis with separation of a solid phase (biochar) from a hydrolyzed phase that was further processed by subjecting it to a two-step fermentation—with a *S. cerevisiae* step followed by an *Escherichia coli* step, similar to the fermentation in the *K. alvarezii* biorefinery described above [142]. The utilization of SWE is a much better use of a green seaweed biomass, especially if considering that HMF is included in the list of the 12 most promising biobased molecules and a potential precursor of pharmaceuticals and bioplastics [143]. Recently, a biorefinery-like process for *U. fasciata* has been developed, thereby separating pigments (total of ~3% of the biomass dw), starch (13%, dw), lipids (3%, dw), protein (13%, dw), cellulose (11%, dw), and ulvan (22%, dw) [140]. This required quite an elaborate process, entailing several steps of Soxhlet extractions, filtrations, centrifugations, isopropanol extraction, bleaching, and various chemical treatments, under both alkali and acid conditions [140]. While this complexity and the associated technological and economic cost of upscaling bring difficulties, this process separated ulvan, a sulfated polysaccharide with biotechnological potential [144], using chilled isopropanol, 2.5:1, *v*/*v*, at −40 °C for 24 h [140]. Hence, though less studied, there is also significant progress towards a green seaweed biorefinery concept.

##### Biorefinery in Brown Seaweed Species

As to brown seaweed species, which are considered an outstanding source of biologically active components [16,145,146], there are plenty more examples of biorefinery approaches, comprising a large variety of species, such as *Alaria esculenta*, *A. nodosum*, *Durvillaea potatorum*, *Fucus vesiculosus*, *Laminaria digitata*, *S. latissima*, *Sargassum tenerrimum* (and other species of the same genus) or *Undaria pinnatifida* [147,148,149,150,151,152,153,154,155]. This diversity of case studies offers the possibility of comparing different strategies and technological articulations for achieving a proper and effective biorefinery.

A biorefinery aiming at the production of alginate, fucoidan, and laminarin from *D. potatorum* has been studied, and, in particular, it was found advantageous an acid extraction step before alkaline extraction in order to attain such multiple valuable polysaccharides from a single seaweed feedstock [147]. Indeed, the process was initiated with an acid extraction (up to 0.1 M HCl solution, 1:20 *w*/*v*, at 60 °C for 3 h) for extracting fucoidan, laminarin, and acid-extractable alginate, which were separated from a supernatant after centrifugation, thus leaving a residual biomass still rich in alginate [147]. Moreover, the supernatant was subjected to a selective precipitation with 20%, *v*/*v*, ethanol and 0.5%, *w*/*v*, calcium chloride, followed by overnight stirring at 4 °C and centrifugation, resulting in a pellet rich in calcium alginate, which was further treated and enabled the production of sodium alginate, and in a supernatant rich in fucoidan and laminarin. This supernatant was subjected to precipitation with the addition of twice its volume in ethanol, followed by centrifugation to separate a solid residue and a novel supernatant whose ethanol was evaporated and, finally, freeze-dried, thereby achieving a product rich in fucoidan and laminarin [147]. The residual biomass after the acid extraction was subjected to an alkaline extraction (28%, *w*/*v*, sodium carbonate solution, at 60 °C for 2 h) and centrifuged, yielding a supernatant—the extracted material—rich in alginate, which was then neutralized and precipitated with 10%, *w*/*v*, calcium chloride solution. The precipitated calcium alginate was further processed which led to a second product composed of sodium alginate [147]. The yields were 7%, w/dw, in fucoidan+laminarin, and 37%, w/dw, sodium alginate in two final products. This process may still be improved, since there are various by-products without a proper valorization, for instance, the solid residual after the alkaline extraction.

For two of the most important brown seaweed species, *A. esculenta* and *S. latissima*, important advances in the direction of a multicomponent biorefinery have already taken place using a combination of mild chemical extraction techniques [148]. In this other case-study, besides alginate, fucoidan, and laminarin, a fourth polysaccharide, usually abundant in brown seaweed, cellulose, was separated. The first step was a slightly acidic extraction with diluted HCl (pH 4.5) of fucoidan and laminarin, which were dialyzed for separation [148]. Afterwards, calcium alginate in the solid material was converted first to alginic acid by the action of a 0.2 M HCl solution treatment and then to sodium alginate with 0.2 M sodium hydrogenocarbonate solution. The soluble sodium alginate was washed and precipitated with a strong acidic 3 M HCl solution, and this step was followed by neutralization and further precipitation with ethanol until isolating alginate [148]. On the other hand, the spent biomass (after alginate extraction) was treated with ethanol and water as well as solutions of hydrogen peroxide, sodium hydroxide (NaOH), and HCl, thereby removing multiple types of other components according to their chemical affinities and leaving a final solid residue much enriched in cellulose [148]. This process represents an advance regarding other previous and comparable processes using brown seaweed as starting material, thereby yielding 70 kg of cellulose, 90 kg of fucoidan+laminarin, and 140 kg of alginate per ton of organic matter in the biomass—corresponding to ~70% dw [148]. Nonetheless, these mass balance results mean that there is still a large fraction of wasted organic material, which is chiefly present in the washing waste streams.

Another brown seaweed species that is very important as a source of biologically active compounds, namely laminarin, is *L. digitata*, for which a biorefinery for the production of specialty chemicals, biofuel, and bioactive substances was developed [151]. This biorefinery has a dilute-acid extraction—0.1 M HCl solution (pH 2–2.5) at 70 °C for 1 h—as its first operation. This was followed by centrifugation and by calcium chloride precipitation and another centrifugation for separation of alginate, and then by absolute ethanol precipitation and centrifugation for attaining a fucoidan-rich fraction. The waste liquor resulting from these operations was evaporated and used to prepare fractions with antioxidant and antimicrobial activity [151]. The solid residue was treated with a diluted acid solution (1.5 N H_2_SO_4_ at 121 °C for 24 min in an autoclave), its pH adjusted with sodium citrate buffer, and subjected to enzyme saccharification, thus forming glucose and other simple sugars, which were fermented with *S. cerevisiae* to produce ethanol. However, this biorefinery strategy had shortcomings, as more than 40% of the starting material was not valorized, and there was still fucoidan in the residue, a problem that warrants further optimization to maximize recovery of this polysaccharide [151].

Finally, a fourth and very recent case-study involving *A. nodosum* and *F. vesiculosus* aimed at the development of an efficient ‘green’ biorefinery [154]. This biorefinery strove to go beyond the usual brown seaweed polysaccharides—alginate, fucoidan, and laminarin—and isolate mannitol and protein, two other major components in this biomass. Except for ultrafiltration for attaining a fucoidan-rich fraction and ultrasound treatment in the route for alginate separation, the various separation processes and principles were similar to those already described above [154]. The ultrafiltration permeate was enriched in mannitol, 8–13%, w/dw, in *A. nodosum* and 15–17%, w/dw, in *F. vesiculosus*, and laminarin, 21–31%, w/dw, in *A. nodosum* and 25–36%, w/dw, in *F. vesiculosus* [154]. Though protein was recovered in this approach, cellulose and other polysaccharides were left behind. Hence, a saccharification and fermentation component would be advantageous in this process and help in bringing it closer to a full-fledged biorefinery.

##### Biorefinery in Seaweed

All these biorefinery case-studies covering a significant diversity of seaweed species show that the overarching goal of achieving a full utilization of all major biomass components with a conjugation of maximal valorization, technological viability, commercial profitability, and environmental sustainability is still somewhat distant and requires further research and optimization. Based on LCA, it has been concluded that though seaweed has the potential to become a sustainable raw material for biorefinery purposes, technologies still require maturing and improvements [129]. In fact, other novel approaches, especially concerning the minimization of environmental impacts and innovative processing technologies, are needed for a successful application of the biorefinery concept to seaweed.

#### 3.1.3. Wet Route and Other Sustainable Approaches

##### Wet Route

Precisely for achieving a sustainable and environmentally friendly biorefinery of the seaweed biomass, WR has been proposed as a possible alternative [154]. This approach entails waiving any drying or rehydrating step in the overall process, thus saving expensive thermal energy, which usually also translates into a large carbon footprint in the DR approach [29,36]. It may also be a technologically advantageous approach, since cell disruption operations in fresh seaweed matrix could enhance the release of bioactive compounds [36]. In this respect, it has been advocated that disrupting seaweed cell walls by mechanical and/or non-mechanical methods may be eased with the expected improvement in yields [36].

Ummat et al. (2024) [154] in their biorefinery study also compared the WR and DR approaches by utilizing both dry and fresh *A. nodosum* and *F. vesiculosus*. Namely, these authors reported that the highest amount of fucoidan was extracted from fresh *F. vesiculosus* seaweed using an acidic solution as a separating agent—a conventional treatment. It has been argued that whereas the high moisture content in fresh seaweed helps in extraction by acting as a co-solvent and enhancing solvent diffusion in the algal matrix, in dry seaweed, algal structure becomes compact and difficult for the efficient mass transfer of the biomolecules to be extracted [154]. The drying temperature itself can cause degradation of any thermally sensitive compounds present in the biomass and thus depress final yields. Moreover, it was observed that a lower number of solids in the ultrafiltration retentate obtained from fresh *F. vesiculosus* than in that from dry seaweed, but protein content was higher in retentate from fresh compared to dry biomass [154]. Regarding the potential of WR, it has been demonstrated the feasibility of a process for attaining laminarin along with fucoidan from fresh seaweed [156].

The application of a WR approach to green seaweed has also been tested [157], involving the extraction of both carbohydrates and protein from *Ulva* sp. biomass. This is challenging because protein is a component of the seaweed cell wall, and it is closely associated with the carbohydrate macromolecules, which increase viscosity and limit access to the proteins. For this purpose, protein alkaline extraction (pH 8.5) followed by isoelectric precipitation (pH 4.0) and, alternatively, carbohydrate/ulvan enzymatic hydrolysis has been proposed for achieving a protein-enriched fraction as outcome [157], this second strategy being more successful. These authors also observed that sugar release from fresh seaweed was much higher than from freeze-dried material, thus also suggesting a relative advantage of WR. Another factor against alkaline extraction may be its induction of aminoacyl cross-linking [158]. However, according to Juul et al. (2021) [159], this does not affect its in vitro digestibility. In any case, further research is warranted as cross-links—occurring both between and within proteins—alter protein conformation and hamper access of proteases to peptide bonds, thus curtailing protein digestibility [158]. Indeed, it has been claimed that amino acid bioavailability may be lowered by cross-linking [160].

In general, protein extraction from typical green seaweed biomass involves either targeted protein extraction [158,161,162] or protein concentration in the sequence of other compounds’ extraction [158,163,164]. This second option is often an outcome of choosing a biorefinery strategy and is also applicable to WR. Protein extraction presupposes a first step of physical destruction, by either High Speed Homogenization (HSH) or Screw Pressing (SP) [159], usually coupled with an alkaline solution extraction [165,166] and/or enzymatic hydrolysis [165,167]. Afterwards, in order to achieve a higher protein concentration, isoelectric or heat precipitation are among the most effective solutions [158,168]. Regarding brown seaweed and WR, it has been investigated whether fresh or dried biomass is better as feedstock in a biorefinery [169]. These authors concluded that, for the specific case of bioenergy production and a solid/water ratio of 1:4, the efficiency of extraction from fresh biomass was higher than that from dried biomass, 13.8 vs. 1.3, thus indicating that fresh kelp (*Saccharina japonica*) is a better feedstock than dried seaweed. The removal of inorganic matter, which interferes with the full utilization of the organic component of biomass [170], seems to be favored in a WR approach [169]. Research on WR application to red seaweed has also been carried out, for instance, in a *K. alvarezii* biorefinery that extracted an agricultural bio-stimulant and a semi-refined form of carrageenan [139] (see above in Section 3.1.2). The conducted literature review showed that studies on WR, regardless of the particular seaweed group considered, remain few and sparse.

There are also technological and efficiency problems concerning WR. In fact, the high water content of the biomass along the process may also hinder the diffusion of some chemicals or solvents in the matrix, thereby encumbering or delaying the release of relevant molecules from algal cells [171]. This has led to the application of novel technologies in combination with wet matrices [72]. On the other hand, fresh seaweed is highly perishable as a result of high moisture and water activity levels [157]. For tackling this issue without applying dehydration/drying, alternative storage concepts have been proposed, such as silage [157]. Indeed, this technique was tested, and while minerals, mannitol, and glucose contents were reduced, there was a relative enrichment in protein and alginate [157]. In particular, the lower mannitol content was partly assigned to a possible consumption of mannitol by lactic acid (LA) bacteria grown during storage. Nevertheless, Bikker et al. (2016) [157] were of the opinion that opting for silage is an effective way to preserve the major structural components of seaweed biomass. Therefore, WR may be advantageous with respect to DR, but there are still operational issues that require study (and new ideas) in order to reap the environmental benefits of avoiding energetically expensive processes, such as drying.

##### Application of ‘Green’ Solvents

For achieving environmental sustainability, a choice for the so-called ‘green’ solvents in the various operational instances where a solvent is needed is also a major current trend, and with the potential to deliver positive results [21,172]. This trend has been reinforced by regulations and legislation seeking to reduce solvent emissions [173,174]. A ‘green’ solvent should be a nonflammable and biodegradable substance (and able to be recycled) with no inhalation hazards and displaying a low volatility [174,175]. In addition, any such solvent must have a set of physicochemical properties that enable it to be used in diverse types of extraction methodologies [174]. Finding an ideal ‘green’ solvent is difficult, as the choice is a compromise depending on the process, the particular seaweed characteristics, and the components to be extracted [20].

Such solvents encompass a wide variety of substances, ranging from water to the more complex and specific Ionic Liquids (ILs), Deep Eutectic Solvents (DESs), and Natural Deep Eutectic Solvents (NADESs) [14,176,177,178]. In fact, ‘green’ solvents may be ascribed to three major groups: (i) biobased solvents (with a natural origin and presence, such as water, ethanol, glycerol, eucalyptol, limonene, other terpenoids or vegetable oils, fitting ethyl acetate, isoamyl acetate, and ethyl lactate better in a different group of ‘low toxicity’ solvents); (ii) supercritical solvents (including natural substances, but in an unnatural state, such as CO_2_); and (iii) neoteric solvents (including synthesized substances, such as DES, and naturally existing substances, NADES) [176].

Water can be regarded as the ultimate ‘green’ solvent, since it is cheap, non-toxic, and the friendliest to the environment [14]. It can be applied in a wide variety of seaweed processing operations, such as maceration, decoction, infusion, or percolation. However, water is unsuitable to extract non-polar substances [179]. This can be partially tackled by applying water at high temperature and pressure because its polarity is lower under these circumstances. The downside is the deleterious effect of these conditions upon thermolabile bioactive compounds [179].

Ethanol is also a usual ‘green’ solvent, and it is typically applied together with water in variable proportions in hydroethanolic extractions [176,180]. As an example, a 30% ethanol hydroalcoholic solution has been combined with a novel extractive technology, PLE (see Section 3.2), in targeting polyphenols in red seaweed, and this technical solution has been shown to yield the most efficient extraction of phenolic compounds [180]. Antioxidant and antidiabetic compounds were effectively extracted with a relatively mild process, thereby providing a framework for future industrial applications [180]. There are also cases of higher ethanol ratios in the hydroalcoholic solution [181]. These authors used a Timatic extractor—a solid–liquid extractor that, in its extraction cycle, alternates a dynamic phase with a static phase for the transfer of extract into the solvent, in winning biomolecules from *Zonaria tournefortii*. The authors used an RSM to conclude that the optimal parameters would be 96% ethanol, 14.4 Timatic cycles, and 20 min sonication [181].

Within biobased solvents, there are also less-known solvents, but exhibiting a significant extraction potential [176]. For instance, Hamiche et al. (2018) [182] used eucalyptol (1,8-cineole, a terpenoid) as a solvent in the preparation of extracts from *Z. tournefortii*. The eucalyptol extracts were distinguished by being rich in phlorotannins. There was an additional advantage in the possibility of solvent recovery through recycling by steam distillation [182]. However, it was found that the phenolic content and antioxidant properties of the ‘green’ extract attained with eucalyptol were not so favorable for future applications as those of a conventional extract (e.g., higher Half Maximal Effective Concentration, EC_50_, 140 vs. 58 μg/mL) [182]. Indeed, the efficiency and yields of these biobased solvents are frequently unsatisfactory, and this represents a major hurdle to their application by the industry. For instance, Savira et al. (2021) [183] compared several solvents with respect to the extraction of fucoxanthin, a carotenoid, from a brown seaweed (*Sargassum duplicatum*) and found that extracts attained with ethanol and ethyl acetate were less antioxidant than those obtained with methanol, a solvent that poses safety risks and to be avoided.

Supercritical fluids have also been explored and are considered promising as alternative ‘green’ solvents for extracting variable components from seaweed [184]. Examples of application of supercritical CO_2_ as a ‘green’ alternative extracting agent include the brown seaweeds *Dictyopteris polypodioides* [185] and *Fucus serratus* [186] or the green seaweed *Ulva flexuosa* [187]. Since these solvents involve the application of a relatively novel technology to seaweed biomass, SFE, more details are given below (see Section 3.2).

Within neoteric ‘green’ solvents and regarding their application to seaweed biomass, IL utilization has been restricted to a few studies [62,188]. The combination of IL and mild extraction conditions is relatively recent and circumscribed to very few studies, such as phycobiliproteins from the red seaweed *Gracilaria* sp. [189] or protein from the green seaweed *Ulva lactuca* [62]. Synthetic IL, such as 1-butyl-3-methylimidazolium acetate (BMIA), 1-ethyl-3-methyl-imidazolium dibutyl phosphate (EMIDP), 1-butyl-3-methylimidazolium dibutyl phosphate (BMIDP), 1-butyl-3-methylimidazolium chloride (BMIC) or choline chloride (ChC), have been tested, for instance in *U. lactuca* [62]. It was reported that EMIDP was strongly selective to proteins, enabling extraction yields up to 80% for proteins and 30% for carbohydrates [62]. Additionally, gel electrophoresis showed that the native structure of the extracted protein was maintained during the whole process, which proves its relative mildness. It has been found that, after optimization of all operational parameters, a maximum extraction yield from *Ulva* spp. of 5.96 mg chlorophyll/g dw was attainable with tributyltetradecylphosphonium chloride (TBTDPC)—a surface active IL [190]. Martins et al. (2021) [190] concluded the possibility of the development of a cost-effective process without compromising the stability of the final product. In fact, the main hurdle for the utilization of IL is the associated high economic cost [191] and remaining uncertainty regarding toxicity [62]. Sequeira et al. (2021) [153] used tetramethylammonium hydroxide (TMAH) for the extraction of alginic acid from brown seaweeds belonging to the genus *Sargassum*. The process delivered promising results and encompassed an acid treatment followed by the application of TMAH [153]. The application of IL to red seaweed can be exemplified with the extraction of carrageenan from *K. alvarezii* [192]. Results showed that the carrageenan extracted through this process had worse gel strength and viscosity, but a higher emulsification index than that of the conventional process [192]. According to these authors, though monosaccharide composition was similar to the usual carrageenan, antioxidant activity was low as a result of decreased sulfate content. Evidence of thermal degradation and IL dissolution was found in the profiling of molecular weights, but this was claimed to enhance bioavailability and functional properties of the produced hydrocolloid [192].

Beyond IL, there is the group of DES, which are fluids composed of two or three substances that can interact through hydrogen bonds in a way that results in a eutectic effect (with melting temperature below that of the individual components). Just as IL, DESs have already been used in the extraction of seaweed polysaccharides and bioactive compounds [193]. Despite the effectiveness of DES (and IL), there is a current trend toward aqueous solutions of DES (and IL) and away from concentrated solutions [193]. It has been claimed that hydrated DES may be better at extracting carrageenan from *K. alvarezii* than non-hydrated DES, highlighting the importance of solvent hydration in optimizing such extractive processes [194]. Nonetheless, it should be noted that some components may present relevant risks [193,195].

Hence, alternative neoteric solvents that already exist in nature (and in foods) may be a better choice due to the absence of unforeseen toxicological aspects. Indeed, NADES may be preferable, given their natural origin and the fact that they have already been tested to some extent in seaweed [176,196]. For instance, phenolics from powdered seaweeds (*Gelidium corneum*, a red seaweed, and *Sargassum muticum*, a brown seaweed) have been extracted using maceration combined with NADES [196]. They reported that the combination of L-LA and fructose (5:1) was the most effective, enabling a final extract phenolic concentration of 2099 mg Gallic Acid Equivalent GAE/L. Moradiya et al. (2024) [193] used neoteric solvents that are naturally present in biological systems—choline glycolate, choline acetate, choline formate, and their DES counterparts, choline chloride/glycolic acid (1:2), choline chloride/acetic acid (1:2), and choline chloride/formic acid (1:2)—in the extraction of alginate from *Sargassum tenerrimum*. It was observed that a hydrated IL was more effective than its non-hydrated form, being the yield maximized up to 54%, which exceeds the equivalent value in a conventional approach [193]. Moreover, physicochemical and rheological features of the alginate extracted by IL compared well with the alginate produced by a conventional method [193].

In any case, there are still big challenges hampering the widespread utilization of ‘green’ solvents. In many instances, the unsatisfactory or less favorable results in comparison to conventional solvents may be ascribed to the novelty of ‘green’ solvents and the dearth of optimization experiences. The convergence to optimal operational parameters can be accelerated by using time- and test-sparing strategies, being RSM quite useful [181,197]. This and other tools may help in addressing the technological viability challenge. Other technical innovations, including innovative extraction technologies (see Section 3.2), or simply new ideas, such as adding water to NADES for reducing the viscosity of these neoteric solvents and enabling a higher extraction efficiency as a result of accelerated mass transfer phenomena [196], may provide solutions to the challenges posed by such novel and untested ‘green’ solvents. Other issues may be related to the commercial viability of the ‘green’ solvents. Namely, the high costs of some neoteric solvents may be addressed by synthesizing new ILs from inexpensive raw materials [198] and by putting in place technologies—based on phase induction, adsorption, or membrane processes—that allow solvent recovery and reutilization [62,199].

### 3.2. Novel Extractive Techniques

#### 3.2.1. General Overview

The conventional extraction techniques have several limitations and drawbacks, especially when applied to difficult matrices, as is the case with most seaweed species. These problems were already mentioned in this review and can be succinctly summarized as the following: (i) loss of valuable components (especially thermolabile compounds) and associated low quality of the attained products (sometimes with low bioavailability of key compounds); (ii) poor extraction yields (often a consequence of inefficient cell wall disruption); (iii) insufficient valorization of the whole biomass (a departure from the biorefinery concept); (iv) lengthy and overly contrived processes; (v) high costs (especially in the case of techniques with high energy demand); (vi) environmentally unsustainability (including a large carbon footprint and hazardous waste); and (vii) toxicological risks to the final consumer [22,68,118,125,200]. In fact, only an adequate extraction technique is able to ensure that the attained components are biologically active, presenting all desired properties and maximizing potential applications [22]. Since this main purpose fails with many conventional methodologies, various advanced extraction methodologies have been developed to efficiently isolate and concentrate valuable components not only from seaweed, but also from other natural resources with identical problems [22]. The level of advancement and technological maturity of these methodologies varies widely, with some already well known and used by the industry, and others classified as emerging or even seen as novel concepts undergoing first experiments. Moreover, selecting one of these techniques depends on the raw material and targeted compounds in the extraction, and it may require specific preparation steps and particular care in the previous preservation of the seaweed biomass [201].

For polysaccharides, the most commonly used novel technologies addressing polysaccharide extraction encompass EAE, MAE, PEF, PLE, and UAE [24,126,202,203,204,205,206,207,208,209]. These cutting-edge methodologies may be combined for better results [210,211] or coupled with more conventional approaches [212]—for instance, hot acidic extraction [213]. In any case, they can also be applied to especially difficult algal matrices [214]. Though these technologies have fewer disadvantages than conventional ones—to which they are usually compared [215,216]—, they are not exempt from problems or limitations [206]. In particular, EAE, which has been advocated as suitable for polysaccharide extraction from seaweed through its effective action in hydrolyzing and solubilizing carbohydrates [217], may fail in a large-scale industrial setting due to unstable enzymes [208]. It should also be remarked that these and other technologies are usually applicable to all three major groups of seaweed (green, red, and brown seaweed), but some techniques may be more apt for particular groups and targeted components [218]. In order to improve the cost-effectiveness of usual extractive processes—usually aiming at a hydrocolloid polysaccharide—, the conception and development of biorefineries is a promising approach because it enables a proper valorization of a larger share of the biomass, thereby combining different—either novel or conventional—technologies and reducing production costs [24,219,220]. It should also be remarked that there are processes that are viable at a laboratory scale, but not at an industrial scale, for instance, grinding in liquid nitrogen [221], thereby dividing techniques into up-scalable and non-up-scalable ones.

Whenever a novel extraction technique is applied for the first time in a particular matrix, the performance of RSM or other optimization methodologies is of critical importance for a more rapid convergence toward the best operational parameters and maximization of extraction yields, in that it spares time and resources by reducing the number of necessary trials [85,204,207,213,216,222,223]. Moreover, by modeling key operational parameters, such as biomass/solvent ratio, temperature, time, and others [224], simultaneously, these tools for experimental design enable valuable insight into the interactivity between factors and possible underlying mechanisms that decisively determine the outcome of an extractive process. In particular, RSM has been widely employed for novel extraction techniques and seaweed [85]. For instance, RSM was decisive in optimizing extraction yields of antioxidants through SWE and UAE applied to brown (*Eisenia bicyclis*, *F. vesiculosus*, *H. elongata*, *U. pinnatifida*), green (*Codium tomentosum*, *U. lactuca*), and red (*Chondrus crispus*, *G. gracilis*, *P. palmata*, *Porphyra dioica*) seaweed species [85].

Another useful approach is to employ techniques applied to other matrices, including non-algal ones, and consider these examples as case-studies and starting points for adjustments and adaptations. Namely, for EAE, despite some enzymes not being available for industrial-scale production, it is important to make use of appropriate and stable enzymes [208], given their substrate specificity and the variable chemistry of seaweed, thereby always requiring adjustments in the choice of enzymes whenever a new matrix is explored [225,226]. Indeed, there have been significant inroads made towards the isolation of new enzymes from marine biota, in particular for the hydrolysis of polysaccharides, such as agarases, carrageenases, glucuronan-lyases, laminarinases, porphyranases, and ulvan-lyases [226]. While for a red seaweed, a carragenase may be of paramount importance in extracting bioactive components, a green seaweed with ulvan may benefit from an ulvan-lyase [227]. Other more common enzymes, namely amylases, cellulases, and xylanases, may also be helpful [227].

Extraction techniques may be divided into three [212] or, possibly, four—if fermentative methodologies are considered as a separate group—broad categories: physical, chemical, enzymatic, and biological techniques. Accordingly, they were grouped together and discussed in more detail in separate sections (see below).

#### 3.2.2. Technologies Based on Physical Processes

Several innovative technologies are based on physical processes and represent progress from the simple and conventional mechanical processes. Namely, HHP (High Hydrostatic Pressure), MAE (Microwave-Assisted Extraction), PEF (Pulsed Electric Field), PLE (Pressurized Liquid Extraction), SFE (Supercritical Fluid Extraction), SWE (Sub-critical Water Extraction), and UAE (Ultrasound-Assisted Extraction) belong to this group of extraction techniques that have been applied to seaweed biomass [8,16,19,22,23,24,25,126,204,205,206,207,209]. They encompass a wide variety of physical processes, involving radiation (MAE), mechanical waves (UAE), electric phenomena (PEF), or pressure–temperature changes (HHP, PLE, SFE, and SWE), and aim at disrupting the algal cell wall (Table 2).

##### Microwave-Assisted Extraction

Among the technologies based on physical processes, MAE is one of the most utilized and studied as an alternative to conventional techniques in the case of seaweed biomass [21,23]. The principle of MAE is based on the exposure of the biomass to electromagnetic waves in the microwave zone of the spectrum, 300 MHz–300 GHz [252]. The energy associated with these waves is dissipated by matter in the form of heat through multiple mechanisms, resulting in a very rapid conversion to heat that is volumetrically diffused and ensuring a very fast temperature increase that can speed up extraction processes [252]. The generated heat causes evaporation of the intracellular fluids, an increase in pressure, and the consequent cell breakage and release of intracellular components into the extracting solvent [206]. With respect to conventional techniques, MAE reduces solvent consumption and enhances efficiency, making it easy to couple MAE with other extraction techniques. Its main drawback is the risk of degradation for thermolabile substances [98,253].

The extraction temperature achieved by application of MAE may vary between 38 and 168 °C (90–100 °C is usual), thereby requiring a power between 500 and 1000 W during a few minutes (or, less usually, longer). In addition, the MAE technique is commonly paired with polar solvents, such as water, acidic and alkali solutions, IL, and DES [206,231,236,237,254,255,256,257]. In particular, it has been studied a water-MAE process for extracting polysaccharides from the brown seaweed *Sargassum thunbergii* [256]. The obtained extract was characterized and contained ~33% of carbohydrates, ~2% of protein, and ~15% of sulfates. The choice of acidic aqueous solutions as extracting media instead of solely water may be justified by a higher overall solubilization rate and more effective extraction, but specifically for polysaccharides, yield may decrease with acidity as a result of their hydrolysis to oligosaccharides and monosaccharides [236]. In any case, these authors managed to effectively extract sulfated polysaccharides from the green seaweed *Ulva prolifera* by MAE. Furthermore, functional property analysis indicated that properties depended on the MAE operational parameters [236]. Indeed, while polysaccharides extracted with a 0.05 M HCl MAE targeting a temperature of 90 °C had the best water-holding capacity and oil-holding capacity, polysaccharides extracted with a 0.1 M HCl MAE and a higher temperature of 150 °C displayed higher antioxidant activity [236]. Therefore, thermal treatment mediated by MAE did not cause a negative effect upon this key set of properties. In the same vein, a study on the brown seaweed *A. nodosum* subjected to various extracting techniques [255] showed that, though conventional technology delivered a higher extraction yield, 0.01 M HCl MAE (90 °C) led to a polysaccharide fraction (fucoidan) with better physicochemical properties, encompassing fucose and galactose contents, sulfate levels, molecular weight, and dispersity index. Regarding these aspects, conventional techniques as well as UAE and EAE underperformed MAE. Another comparative study [231] assessed 0.1 M HCl MAE (up to 92 °C) and UAE and found that the antioxidant properties did not exhibit any clear trend or large improvements by using either MAE or UAE. Zayed et al. (2023) [237] explored fucoidan extraction from different brown seaweed species (*F. vesiculosus*, *Fucus spiralis*, and *Laminaria saccharina*) by MAE. After refinement of operational conditions, MAE was carried out using a ratio of biomass: 0.1 M HCl aqueous solution (containing 2 M calcium chloride, CaCl_2_) of 1 g to 25 mL for 1.0 min [237]. Using these same extraction conditions, chemical characterization showed a substantial heterogeneity of outcomes. In comparison to conventional solvent extraction, MAE resulted in lower molecular weight polysaccharide products [237].

A paradigmatic example of MAE application to red seaweed involves carrageenan extraction from *Solieria chordalis* [258]. The novel technology was compared to a conventional one, and different KOH concentrations in the aqueous extracting medium were trialed. Boulho et al. (2017) [258] concluded that alkali addition and concentration were deleterious, thus choosing a water-MAE at 90 °C for 10 min, which allowed a carrageenan yield in excess of 29%, w/dw, a value that also surpassed that of the conventional technique. Another study provides an example of extraction optimization involving MAE and phenolic compounds (as well as antioxidant activities) [80]. Extracts from the brown seaweed *Sargassum vestitum* were prepared by using RSM with Box–Behnken Design (BBD) [80]. These authors found that solvent concentration was the most influential factor on yields and antioxidant levels, followed by microwave irradiation time and power.

As a contribution to a biorefinery strategy, Sasaki et al. (2024) [152] used seaweed waste (rhizoid and stem) of *U. pinnatifida* as raw material in a continuous water-MAE procedure for the production of protein and fucoidan in two MAE steps. The first MAE treatment at 100–120 °C was optimal for recovering protein and peptides, and the second MAE at 150–160 °C was the best for fucoidan extraction. Indeed, the highest fucoidan yields were obtained with water-MAE at 150 °C in 30 min, reaching 12.3%, w/dw [152].

On the other hand, MAE can also be tested with more complex extracting media or non-purely aqueous solvents [254,257]. A two-phase (ethanol and ammonium sulfate aqueous solution) MAE has been applied to the extraction and separation of polysaccharides from *Sargassum pallidum* [254]. For 21%, *w*/*w*, ethanol and 22%, *w*/*w*, ammonium sulfate, optimal extraction conditions were found for a biomass/solvent ratio of 1:60 g/mL, extraction time of 15 min, power of 830 W, and temperature of 95 °C [254]. Under these optimal conditions, polysaccharide extraction yields for the top and bottom phases were ~0.8% and ~6.8%, w/dw, respectively. Cao et al. (2018) [254] measured a stronger α-glucosidase inhibitory activity in the top phase polysaccharides than those present in the bottom phase, suggesting a potential antidiabetic activity. A DES-water extracting medium was conjugated with MAE to recover polysaccharides from *F. vesiculosus* [257]—an instance of a ‘green’ solvent presented below (see Section 3.2.5).

Very briefly, MAE is advantageous in being rapid, requiring minimal solvent consumption, and preserving compound integrity [21]. Based on its application to seaweed biomass in recent years, MAE has shown to be effective in extracting a variety of molecules, including polysaccharides, phenolics, and other antioxidants [259]. It is still an evolving technique that needs further refinement of conditions and optimization of parameters—for instance, it has been shown that higher power can increase extraction yields [260]—, but clearly with a large potential for the seaweed processing industry and other sectors benefiting from seaweed component applications.

##### Ultrasound-Assisted Extraction

The UAE technique is based on the physicochemical principle of acoustic cavitation and may produce a wide gamut of phenomena depending on its duration, frequency, and other parameters [261]. Specifically, ultrasound waves with a frequency between 20 kHz and 100 kHz are generated in the solvent, leading to the production of bubbles and low- and high-pressure zones [204,262]. The growth and collapse of these bubbles cause sound waves to be converted into mechanical energy, thus inducing damage to the seaweed cell walls. It should be noted that besides fragmentation and erosion—which may pose problems—, ultrasounds promote sonocapillary—a heightened penetration of solvent into the pores and canals of the seaweed matrix [261]—and sonoporation—an augmented cell membrane permeability that contributes to release intracellular components into the extraction medium by opening membrane pores [263]. Relatively mild UAE conditions, especially if low frequency and short exposure time with adequate intervals between cycles for cooling are applied, may avoid the problems of fragmentation and erosion in the seaweed matric and promote optimal sonocapillary and sonoporation. Moreover, UAE operates at low temperatures, which ensures the preservation of thermolabile compounds [206].

Main solvents used as extractive medium in UAE applied to seaweed biomass are water and acidic solutions, with other solvents also a possibility [215,246,264]. A relevant study relates to UAE of carrageenan from the red seaweeds *Euchema denticulatum* and *K. alvarezii* and alginate from the brown seaweeds *Sargassum binderi* and *Turbinaria ornata* [246]. The effect of crucial operational parameters (temperature, pH, biomass/water proportion, ultrasound power, and duration) on UAE performance and outcomes has been investigated [246]. These authors were able to extract polysaccharides equivalent to up to 55%, w/dw, of the biomass, thereby requiring only 15–30 min. Another relevant brown seaweed polysaccharide with large industrial interest is laminarin, which may be extracted from *A. nodosum* and *Laminaria hyperborea*, and whose extractability by an acidic solution UAE was tested by Kadam et al. (2015) [84]. Given the available levels of laminarin in these species, yields were quite satisfactory, reaching approximately 6%, w/dw, for both species [84]. Furthermore, the antioxidant activity of both extracts, as measured by 2,2-DiPhenyl-1-PicrylHydrazyl (DPPH), was high, reaching inhibition levels of more than 87%, and antimicrobial activity was also detected [84]. On the other hand, Santos et al. (2025) [85] tested UAE as well as SWE and a conventional SLE in the extraction of antioxidants from ten seaweed species and achieved modest results with aqueous UAE. Though UAE outperformed SLE in most cases, it was concluded that SWE delivered better results than UAE [85]. Brown seaweed *E. bicyclis* extracts were the most antioxidant, but while 2,2′-Azino-Bis(3-ethylbenzoThiazoline-6-Sulfonic acid (ABTS) index varied in UAE extracts from this seaweed between 51 and 58%, it reached >64% in SWE extracts [85]. Moreover, Brain-Isasi et al. (2022) [265] studied the simultaneous recovery of agar and phycobiliproteins from the red seaweed *Gracilaria chilensis* and attained satisfactory results (45% recovery of total phycobiliproteins and ~25%, w/dw, of agar yield after phycobiliprotein extraction). However, Brain-Isasi et al. (2022) [265] stated that there was still margin for improvement by optimizing buffer/biomass ratio or the freeze–thaw effect.

Regarding UAE operational parameters, different times and temperatures may be tested [206,246] as well as variable ultrasound frequencies [255,266,267]. However, lengthy operational times and temperatures higher than room temperature are not advisable and represent a loss of the relative advantage of the UAE technique with respect to conventional technologies, such as SLE. Concerning frequency, though 20 kHz is the most applied level [255,265], higher frequencies have been experimented [266,267]. In particular, while Hmelkov et al. (2018) [266] extracted fucoidan from the brown seaweed *Fucus evanescens* and used water and a frequency of 35 kHz, Rahimi et al. (2016) [267] recovered ulvan from the green seaweed *Ulva intestinalis*, thereby applying a 53 kHz frequency. In this study, four other independent UAE operational parameters were trialed, extraction temperature (50–90 °C), extraction time (20–40 min), water/seaweed biomass ratio (50–70), and pH (7.0–9.0), and optimized conditions for better yields were found, an UAE operation at 66 °C for 40 min and with pH 7.0 water/seaweed biomass ratio of 50. Ultrasonic power has also been studied [268]. These authors, after testing power levels between 100 and 300 W with a water/biomass ratio of 30 mL per g, observed a higher extraction of pigments (chlorophylls and carotenoids) from *U. pinnatifida* with 300 W. However, a higher ultrasonic power is not always conducive to higher extraction yields, since, for instance, in chlorophylls, too much power may lead to decomposition through the associated thermal effect [268]. A step further beyond operational parameter testing and optimization is to model kinetically UAE [269] for the extraction of fucoxanthin from the brown seaweed *Sargassum fusiforme* with ‘green’ or ‘low toxicity’ solvents. In fact, Nie et al. (2021) [269] found an optimal second-order kinetic model, with rate constant, equilibrium level, and initial extracting rate as parameters, for dynamic UAE under different operational conditions. A solvent/biomass proportion of 40 mL per g, 75 °C for 27 min with 53% amplitude, and ethyl lactate as solvent were identified as optimal parameters [269]. Finally, regarding the UAE, it should be remarked that, given its articulation flexibility, it aptly suits integration into larger biorefinery processes [154].

As an overall assessment, it can be mentioned that the UAE is one of the most promising novel techniques due to its many advantages, such as high yields, limited solvent consumption, rapidity, preservation of biomass components from thermal degradation, flexibility to articulate with other techniques like MAE, and high ‘greenness’ scores [85,117,204]. However, the high energy input required to perform UAE at an industrial scale is a drawback.

##### Pulsed Electric Field

Regarding PEF, it essentially aims at an increased permeability of algal cell membranes [126], but it achieves this by electrical field means instead of using acoustic cavitation as UAE. The induced permeability by the application of electric field pulses (electric field strength of 10–50 kV/cm, but also with lower levels) through electrodes bracketing the sample, also called electroporation, has been employed to extract various intracellular components, ranging from water and ions to proteins and secondary metabolites [126,204,270]. The success in applying PEF depends on several factors, including electric field strength, specific energy input, number of pulses, and temperature [271]. Its application to seaweed is still quite limited, being suggested by Robin and Golberg (2016) [272] as a technological component of a larger seaweed biorefinery process (see Section 3.1.2). In any case, it was possible to use PEF for specific protein extraction from *Ulva* sp. [273]. In another study on green seaweed and protein extraction [167], it was shown that PEF may be effective in protein extraction from *Ulva* sp. This technology can also target more specific compounds, such as the phycobiliprotein—composed of a protein part bound to a phycobilin chromophore—β-phycoerythrin in the fresh biomass of the red seaweed *Porphyridium cruentum* [274]. These authors studied the electric field strength in the 2 to 10 kV/cm range, and pulse duration of 30–150 μs. A value of 32 mg/g dw of β-phycoerythrin in the extract after 24 h of treating *P. cruentum* cells at 8 or 10 kV/cm for 150 μs has been determined [274]. It was noted that β-phycoerythrin release was not immediate after the PEF treatment, but, in most cases, was completed only after 6 h, thereby suggesting that, besides β-phycoerythrin diffusion across the cell membrane, dissociation of this compound from the cell structures was a determining factor in the extraction [274]. Furthermore, a study on polyphenol, flavonoid, and carbohydrate extraction from three seaweed species, *A. esculenta*, *P. palmata*, and *U. lactuca*, showed similar outcomes to those obtained with conventional hot water extraction [275]. The advantage of PEF to this conventional technique lies in its non-thermal nature and rapidity [275].

It may be concluded that in comparison to other processes, the PEF technique does not require the addition of chemicals and has a low energy and water consumption [276]. From an industrial point of view, this physical technique has additional important advantages, such as its rapidity (from a few seconds to minutes), mild conditions (in particular, concerning temperature), possibility of application to wet seaweed biomass (WR, see Section 3.1.3), high food safety levels, and up-scalability [276]. In fact, PEF is already being exploited in the food industry [158].

##### High Hydrostatic Pressure

As its name suggests, HHP is a technology based on high pressure (thousand times higher than atmospheric pressure, usually in the 200–600 MPa range) that is applied by a liquid upon a matrix, thereby leading to deprotonation of charged groups and disruption of weak bonds in cell membranes, which, in turn, enhances cell permeability and extraction yields [24]. This technique has already been applied to seaweed biomass [277,278,279,280]. The application of HHP to *S. muticum* has been assessed in terms of total extracted sugars, antioxidant activity, and overall yield [279]. The outcomes of the HHP extraction were positive in that the yield varied between 320 and 400 mg/g dw with 3.6–4.8-fold enhancement for total sugars in comparison to a conventional technique [279]. These authors reported that optimal conditions for HHP were attained with 5–5.5 min treatment time and 3000 bar pressure. Positive results were also achieved for red seaweed (*P. palmata* and *S. chordalis*) and various phytochemicals, including protein [280]. Precisely, O’Connor et al. (2020) [278] tested whether HHP (6000 bar applied for 4 min) was helpful in protein extraction from two brown seaweed (*F. vesiculosus* and *A. esculenta*) and two red seaweed (*P. palmata* and *C. crispus*) species. A favorable and comparatively advantageous—with respect to autoclave processing—effect of HHP treatment on protein extraction was identified in the specific case of *F. vesiculosus*, with ~24% of total protein recovery [278].

Although HHP may be suitable for extracting thermolabile substances, pressure-induced protein denaturation—and conformation changes in general—are a serious possibility [277]. Another main obstacle for HHP is the sheer levels of high pressure that are required and the resulting large investment costs, especially if upscaling to an industrial level. Accordingly, further research and technological advancement in the production of cheaper equipment are warranted.

##### Pressurized Liquid Extraction

Another technology that uses high pressure is PLE, also known as accelerated solvent extraction, which comprises the specific case of water as solvent, commonly referred to as SWE and treated separately below [27,206]. The technology uses a variety of solvents at high temperature (usually in the 50–200 °C interval) and pressure (often in the 35–200 bar range), while maintaining them in the liquid state [117,281]. There are not many examples of PLE applications to seaweed. Namely, the brown seaweed *S. japonica* was treated with a deep eutectic solvent (choline chloride/glycerol in a 1:2 ratio) combined with subcritical water, and the recovery of alginate and fucoidan from this biomass was studied [282]. The potential of PLE was also highlighted by Fayad et al. (2017) [283] for the extraction of cosmetically valuable anti-hyaluronidase from the brown seaweed *Padina pavonica*, yielding positive results—even in comparison to other innovative techniques and involving the testing of ethanol, ethyl acetate, petroleum ether, and water as solvents—for an optimal set of operational parameters, two 60 s cycles at 60 °C and 150 bar. Additionally, the lipid fraction in two green seaweeds (*U. intestinalis* and *U. lactuca*) and four brown seaweed (*F. vesiculosus*, *Dictyota dichotoma*, *Cystoseira baccata*, and *H. elongata*) species has been investigated, and the antioxidant and antibacterial activity of ethanolic PLE extracts has been evaluated [284]. Otero et al. (2018) [284] found that *F. vesiculosus* ethanolic PLE extract had a higher antioxidant activity (50% DPPH inhibition, IC_50_, with only 7.17 μg/mL) than the other seaweed species. In a comparison across solvents (hexane, ethyl acetate, acetone, ethanol, and ethanol/water 50:50, *v*/*v*) at three set temperatures (80 °C, 120 °C, and 160 °C) and 100 bar pressure in PLE, ethyl acetate, a ‘low toxicity’ solvent, enabled a better extraction of long chain fatty acids (oleic acid, arachidonic acid, and eicosapentaenoic acid) with ω6/ω3 ratio near 2.7—the lowest ω6/ω3 ratios were achieved with the more polar solvents, ethanol and ethanol: water 50:50, 2.2 and 1.9, respectively [284].

Therefore, the utilization of PLE may be advantageous in that it requires less solvent and shorter extraction times than conventional methods without compromising high efficiency [103,206]. On the other hand, the application of high temperature is troublesome because it may decompose thermolabile molecules; its expensive equipment and high energy consumption are also unfavorable aspects, which limit its up-scalability, and it lacks selectivity [24,27].

##### Sub-Critical Water Extraction

The SWE technique—a particular instance of PLE—involves the utilization of water subjected to temperature-pressure ranges (100–374 °C and 10–221 bar) that keep water liquid phase even though above the normal boiling point of water [16,285]. This technology enables faster extraction rates, reduced solvent consumption, and higher yields in comparison to conventional techniques [8]. It is also a readily scalable methodology, since it is not too demanding in terms of operational pressure ranges [16]. Temperature, pressure, extraction time/flow rate, and particle size are key parameters of operation in SWE, whose application into seaweed biomass can be analyzed in accordance with a set of the five stages: (i) wetting of the biomass with subcritical water; (ii) desorption of targeted compounds from the solid matrix, also comprising the breakdown of chemical bonds; (iii) dissolution of these compounds in subcritical water; (iv) compound diffusion from the biomass matrix into the surface; and (v) mass transfer of the compounds from the surface into the subcritical water bulk [16]. The high temperature used in SWE lowers water viscosity and surface tension, thereby increasing diffusivity, enabling deep solvent penetration into the biomass, and powering mass transfer. Moreover, high temperature also weakens intermolecular forces, helping in the release of compounds from the biomass [286]. However, this can be a problem if both targeted and undesirable compounds are rendered more soluble, thus generating a less selective process, and whenever there are thermally labile components [16]. On the other hand, high pressure is relevant to biomass wetting and breaking down cell wall structures, thus improving extractive yields. However, high pressure also leads to high operational costs and hinders upscaling [16]. Particle size may also be influential, particularly regarding mass transfer rates, as smaller particles provide larger surface area per unit mass and have shorter internal diffusion paths [287].

Within seaweed, the main field of application of SWE has been the extraction of biologically active components from brown seaweed species [16]. Overall crude extract yield may vary extensively as a function of the particular species and operational parameters. Namely, it has been reported that approximate yields of 19%, w/dw, in *Hizikia fusiforme* and 36%, w/dw, in *L. japonica*, but over 62%, w/dw, in *U. pinnatifida*, were obtained under the same SWE conditions (210 °C, 30 bar, and a water/biomass ratio of 20 mL/g) [288]. On the other hand, a higher crude extract yield, ~76%, w/dw, after submitting *Ecklonia maxima* to an SWE involving a combination of 180 °C, 40 bar, and a water/biomass ratio of 30 mL/g has been observed [243]. At a more detailed level, it is possible to distinguish between a lower temperature range (<160 °C), more favorable to high polysaccharide yields, and a higher one (>180 °C) that enhances overall, total phenolic, and phlorotannin yields as well as antioxidant activity in the attained SWE extracts [16,244]. Even more specifically, Gan and Baroutian (2022) [244] identified an optimal fucoidan yield at a lower temperature level, 120 °C. For antioxidant compounds, Santos et al. (2025) [85] reported an ABTS index in SWE (at 190 °C) *E. bicyclis* extracts, >64%, that exceeded those attained in UAE and conventional SLE extracts from the same seaweed.

In summary, the SWE methodology is able to efficiently extract key components from the seaweed biomass and to be used in a selective manner by modifying process conditions (temperature and other operational parameters). This is achieved while minimizing extraction time and consumption of hazardous chemicals in comparison to conventional methodologies. It is also important to note that though laboratory-scale experimental work is essential for testing SWE, it fails to capture the impact transport phenomena effects have on overall reaction time and operational parameters at a larger industrial scale [16].

##### Supercritical Fluid Extraction

Whenever pressure–temperature binomials go beyond certain thresholds, known as critical points and always dependent on the particular substance—being 374 °C and 221 bar for water and 31 °C and 73.8 bar for CO_2_—, the pressure–temperature curve indicating conditions under which liquid and gas phases can coexist ends and, at higher temperatures, the gas comes into a supercritical phase. Precisely, the utilization of solvents in a supercritical phase is the basis of the SFE technique. In particular, application of SFE with CO_2_ as supercritical fluid to extract targeted compounds is a promising route for valorizing seaweed biomass while reducing solvent consumption and extraction time and enhancing selectivity with respect to conventional techniques [85]. Besides being less demanding in terms of pressure–temperature, CO_2_ as supercritical fluid has various advantages, such as its non-toxicity, cheapness, and recyclability [289,290]. It should also be noted that it is inherently non-polar with low dielectric properties, making it perfect for dissolving lipophilic apolar molecules. In mechanistic terms, the high diffusion and matrix penetration ability of supercritical CO_2_ leads to shorter extraction times by accelerating mass transfer and, after extraction, it can be easily and quickly removed through depressurization by-passing the costs of solvent evaporation [176]. It has also been tested in seaweed SFE as part of binary and ternary mixtures, namely with ethanol and water [187,240,289,290,291,292].

There are already some relevant studies on SFE application to seaweed [184,185,186,187,240,289,290,291,292,293]. There are examples of SFE and brown seaweed—*D. polypodioides* [185], *F. serratus* [186], *S. japonica* [240], *U. pinnatifida* [291]—, red—*Gracilaria mammillaris* [292], *K. alvarezii* [293]—, and green seaweed—*U. flexuosa* [187]. Using SFE with 60 °C, 500 bar, and CO_2_ flow rate of 24 mL/min, 16 mg of fucoxanthin per g of dry *D. polypodioides* has been achieved [185]. Heffernan et al. (2016) [186] targeted the whole carotenoid fraction in *F. serratus* with somewhat milder conditions (50 °C, ~300 bar, and CO_2_ flow rate of 10 mL/min) and obtained 16 mg of total carotenoids per g dw.

In order to improve CO_2_ solvating properties, a modifier can be added, being ethanol and other ‘green’ solvents preferred over older conventional choices, such as methanol. Recently, Honda et al. (2022) [291], using a binary system of supercritical CO_2_ and ethanol, managed to recover fucoxanthin from *U. pinnatifida* under relatively demanding conditions, 160 °C and 300 bar. Likewise, Fabrowska et al. (2017) [187] combined CO_2_ and ethanol in an SFE, but with milder temperature (40 °C) and 350 bar, targeting total carotenoids in *U. flexuosa*. Ospina et al. (2017) [292] used CO_2_-ethanol in SFE for extracting antioxidants from *G. mammillaris*, achieving best results with 60 °C, 300 bar, and 8% co-solvent (ethanol) as SFE operational parameters. The results showed the viability of using SFE for attaining antioxidant extracts from *G. mammillaris* [292]. Another binary system but composed of supercritical CO_2_ and sunflower oil (co-solvent at 2%), achieved positive results in extracting carotenoids from the brown seaweed *S. japonica* under similar temperature, 55 °C, and 300 bar, thereby recovering 2.4 mg of total carotenoids per g dw [240].

Though CO_2_ SFE is up-scalable and already used in the food industry for various purposes, its applications are limited to lipids and lipophilic substances (carotenoids, groups of phenolics, etc.) due to CO_2_ low polarity—a problem mitigated through combination with more polar co-solvents [126,294]—, it requires high initial investment [176,295], there are environmental concerns related to being a greenhouse gas, and CO_2_ as well as other supercritical fluids display complex behavior in conditions near their critical points [296]. All this shows how more research is warranted to improve the viability of SFE application to extracting valuable components from seaweed.

##### Other Physical Extraction Technologies

Other innovative technologies, whose application to seaweed biomass is less known and characterized by a dearth of scientific literature, are the so-called Instant Controlled Pressure Drop, also known as ‘Détente Instantanée Contrôlée’ (ICPD/DIC) [68] and the Cold Plasma-Assisted Extraction (CPAE) [200,297].

The ICPD/DIC technique was used as pre-treatment for the extraction of carbohydrates, carotene, and other substances from the brown seaweed *S. muticum* [298]. The biomass material was placed in a processing vessel, and a saturated steam pressure of 100 kPa was established, followed by an instant vacuum release, and a final release of pressure to the atmospheric level in the processing vessel. The ICPD/DIC technique improved the extraction of carotene at 20 s under 100 kPa in thalli of *S. muticum* [298]. Another example of an alternative and potentially ‘green’ technique is CPAE. This involves cold plasma as an extracting agent, and it entails the application of an electric or magnetic field and a partially ionized gas (composed of ions and reactive neutral radicals) acting precisely as cold plasma [297]. Plasma technology as pre-treatment to the green seaweed *Chaetomorpha linum* for the purpose of bioethanol production has been tested and applied [299]. The pre-treatment was carried out under atmospheric pressure and at room temperature, being *C. linum* placed in a reactor vessel and properly sealed [299]. The seaweed biomass was subjected up to 60 min to a gas flow rate of 0.01 L/s and the ozone concentration in the gas stream was 1%. For ozone formation, more than 10 kV voltage in the electrodes of a dielectric barrier was necessary to ignite an adequate discharge and generate the corresponding plasma [299,300]. Though plasma technology did not lead to the best outcomes, it was able to significantly contribute to the extraction of a carbohydrate fraction [299].

#### 3.2.3. Technologies Based on Chemical Processes

Conventional processes, such as SLE, largely operate on the basis of chemical interactions and affinities, so the novelty in chemical processes has to lie in the differentiated chemical properties of the extracting medium and in factors such as pH, redox potential, or ionic strength. Technologies involving a solid-phase interaction (SPE, SPME), pH-shift methodology (pHE), and the utilization of ILs as solvents (for ILs and novel solvents with innovative properties, see Section 3.2.5) are all examples of innovative techniques essentially based on phenomena of a chemical nature [20,21,68,249,301,302] (Table 2).

##### Solid-Phase Extraction

The principle underlying SPE is the chemical phenomena of adsorption/absorption and desorption that occur between a solid phase and a liquid medium that can be attained from seaweed biomass. Some specific seaweed components can be stripped from this liquid medium and retained in the solid phase, and afterwards recovered by desorption. The application of SPE and SPME is still mostly confined to analytical purposes, thus placing these techniques in a group of eminently laboratory-scale methodologies [301]. A potentially useful application of SPE that could be envisaged at a larger scale and for extractive and preparative purposes involves its articulation with PLE [271,303]. The PLE-SPE technique was applied to the extraction of phenolic compounds from seaweed-derived (*Porphyra tenera* and *U. pinnatifida*) food products [303]. Concerning SPME, it is an emerging solvent-free sample preparation technique that, just like SPE, is underlain by the principle of adsorption/absorption and desorption. This methodology is seen as an alternative to conventional extraction techniques because it fuses sampling, extraction, isolation, and concentration of seaweed compounds into a single operation [301]. Typically, the solid phase is constituted by small, fused silica fibers coated with a sorbent substance that is selective in accordance with its chemical affinity to the targeted molecule(s). This aspect contributes to the high efficiency of SPME [301]. In general, SPME is considered to be a rapid and economically accessible operation, and it is also possible to reutilize the fiber. However, though it is a technique that uses no organic solvent and fewer pollutants, it is less suited for the extraction of polar molecules or substances displaying low volatility, and the fiber’s frailty may also be a problem [301,304]. Another problem is the availability of adequate stationary phases and fiber coatings, especially considering the large variability and high specificity of compounds in seaweed biomass.

##### pH-Shift Extraction

The application of pHE as seaweed extraction technology is uncommon, and it is more appropriate for specific components, such as protein [249]. In this case, the application of pH-shift coupled with isoelectric point precipitation may enable a maximization of the extraction yield of seaweed protein [250,277]. In particular, Vilg and Undeland (2017) [250] aimed to develop a simple and scalable pHE technique applicable to wet *S. latissima* and investigated crucial operational parameters, such as pH and temperature. These authors determined a maximum protein solubility at pH 12, achieving 34% of total protein extracted with 5.56 volumes of extraction solution. Furthermore, osmoshocking significantly increased the yield [250]. Protein precipitation was possible below pH 4, and the highest precipitation yield, 34.5%, was reached at pH 2. After a pH-shift combining alkaline extraction and acid precipitation, ~16% of the seaweed protein was recovered, which was deemed acceptable, but improvable—if compared to protein extraction from soy—by Vilg and Undeland (2017) [250]. For other components in the seaweed biomass besides protein, pHE performs quite poorly. A total solute release of only ~10% has been reported, with a combination of PEF and pH-shift applied to wet *Gracilaria* sp. [249].

##### Assessment of Technologies Based on Chemical Processes Applied to Seaweed

Some of these chemically novel techniques are still being tested at a laboratory or pilot-scale, and the feasibility of their upscaling is dubious and will require much further investigation and technological development. Moreover, they presuppose the availability of economically accessible materials with high selectivity for specific components in seaweed biomass, which may be difficult to attain. This also means that their application as massive extraction techniques with a broad range of seaweed components being released and solubilized is out of their scope and nature.

#### 3.2.4. Technologies Based on Enzymes and Biological Systems

Enzymes are catalytic proteins synthesized by living organisms that may operate autonomously and, as such, can be integrated in extraction and other processing procedures—specifically in the case of EAE (Enzyme-Assisted Extraction) applied to seaweed biomass. Moreover, full living organisms may also be used as extracting agents, as in the case of seaweed fermentation. A biological system may provide not only an enzyme, but a full operational kit of enzymes and other auxiliary factors, thereby enabling a highly effective disruption of the algal cell wall and release of the inner cell contents (Table 2).

##### Enzyme-Assisted Extraction

The application of EAE to seaweed biomass is one of the main experimental areas in the broader field of novel extraction techniques targeting valuable components in the various seaweed species [16,19,23,68,197,204,206,217,271]. The reason for such large scientific interest lies in the underlying principle and resulting effectiveness of EAE. This technique is based on the fact that enzymes act as catalysts with high selectivity and specificity, as well as on the ability of some specific enzymes to effectively decompose the cell wall of seaweed—itself inspired by the previous experience with plants—, thus releasing entrapped intracellular molecules into an aqueous solution [204,305]. For EAE, the ratio of enzyme amounts to biomass, temperature, pH, particle size, and solvent used are key operational parameters that have to be optimized in order to improve extraction yields [68,110,204]. Commonly used enzymes in EAE include lipases, polysaccharidases, and proteases. The latter two groups are the most frequently employed in extracting bioactive compounds from seaweed [271]. Some of the most often used enzymes target carbohydrates, such as agarase, amylase, arabinase, carrageenanase, cellulase, glucanase, and xylanase [271,306].

Malvis Romero et al. (2023) [228] produced a recent study that is representative of the combination of proteases and carbohydrases. In particular, these authors coupled cellulases and proteases to bring about cell wall disruption and ulvan extraction from the green seaweed *Ulva fenestrata*. The researchers investigated the effect of extraction time on the ulvan yield and its key properties (molecular size, presence of functional groups, purity level, and antioxidant activity), thereby demonstrating that higher extraction times were conducive to higher ulvan yields [228]. Teixeira-Guedes et al. (2023) [217] also used a multi-enzyme complex of carbohydrases and proteases to extract cell contents from the biomass of four seaweed species, comprising brown (*F. vesiculosus*), green (*U. rigida*), and red (*Gracilaria vermiculophylla* and *P. dioica*) species. However, this study endeavored to sequentially apply cellulolytic and proteolytic enzymes to seaweed biomass. It was found that sequential use of enzymes ameliorated overall extraction yield by 30–160% when compared to the control (conventional aqueous extraction) [217]. More specifically, while carbohydrate solubilization increased to 35% in *F. vesiculosus*, 77% in *U. rigida*, 28% in *G. vermiculophylla*, and 66% in *P. dioica*; protein solubilization provided a different relative impact in the studied species, which increased to 42% in *F. vesiculosus*, 52% in *U. rigida*, 55% in *G. vermiculophylla*, and 47% in *P. dioica* [217]. Accordingly, the sequential use of a complementary set of enzymes was shown to be an efficient strategy for extracting fractions with functional potential. Manns et al. (2016) [44] treated the brown seaweed *L. digitata* with a mixture of alginate lyase—an enzyme specific to a polysaccharide that is present in brown seaweed, alginate—and cellulose. All available glucose in the biomass was released within 8 h. In addition, it was observed that application of the cellulase alone released only half of the glucose, indicating that the utilization of alginate lyase is really advantageous [44]. This enzyme seemed to induce the selective removal of alginate, thereby helping cellulase to carry out its degradation of laminarin and cellulose in the biomass [44]. In this regard, it should be noted that the substrate of an EAE enzyme may be different and is often different from the main targeted seaweed component in the extraction process. Studies have demonstrated that a combination of cellulase and xylanase enzymes can improve the extraction of protein, thereby leading to higher yields [307]. Precisely, this has been observed in the extraction of protein from *P. palmata* [307].

Some comparisons across techniques that have been carried out show EAE as disadvantageous [16]. An example concerns fucoidan extraction from the brown seaweed *Nizamuddinia zanardinii*, whose extractive yields varied from 3.6%, w/dw, for UAE to 13.2%, w/dw, for SWE, being EAE (cellulase, β-glucanase, and proteases) intermediate with 4.3–5.6%, w/dw [308]. Regarding laminarin extraction from the brown seaweed *E. maxima* [197], cellulase was used to hydrolyze the seaweed material, and it was found that laminarin extraction was significantly influenced by linear and quadratic effects of pH and temperature. Moreover, in comparison to a conventional dilute-acid thermal hydrolysis (pH 1.0 and 70 °C), EAE outperformed in the release of reducing sugars and in solubilized yield, but not in the selective extraction of laminarin [197]. On the other hand, a study on ulvan extraction from *U. lactuca* compared a conventional technique based on heating with EAE using cellulase and protease and concluded that EAE substantially increased extraction efficiency with respect to the conventional technology, 17.2%, w/dw, vs. 3.0–13.1%, w/dw [309]. A comparison between conventional SLE producing aqueous, hydroethanolic, and ethanolic extracts and EAE involving proteases and cellulases showed EAE as the most efficient process in the cases of a brown (*F. vesiculosus*) and a red (*P. dioica*) seaweed [310]. Overall extraction yields were higher for EAE than for the SLE, and total phenolic content in the *F. vesiculosus* EAE extract was at least 10-fold higher than in SLE extracts, 229.2–311.3 mg GAE/g extract vs. 4.3–19.6 mg GAE/g extract, presenting a good correlation to antioxidant activity, as measured by the ABTS and Oxygen Radical Absorbance Capacity (ORAC) methods [310]. The same general trends were observed in the case of *P. dioica* [310]. Equally noteworthy, EAE outperformed the conventional technique in the extraction of both soluble protein and reducing sugars. Another study on the *F. vesiculosus* and focusing on EAE vs. conventional aqueous methodologies, further buttressed the case of a relative advantage in choosing to apply EAE [311]. Actually, *F. vesiculosus* subjected to EAE brought about extracts with a neutral sugar content that was 34% higher and a reducing sugar content that was 21% higher than the same parameters in extracts obtained by conventional techniques. Concerning plant growth regulators, the concentrations of isopentenyladenosine and cis-zeatin were augmented by 6 times and 28 times, respectively, when applying EAE instead of an SLE [311]. As these examples illustrate, such comparisons are rather difficult because of differences in the techniques that are chosen to be tested and also in the species, targeted compound(s), enzyme(s), and operational parameters used in each technique.

The relative advantages of EAE with respect to conventional techniques lie in its extraction efficacy, high specificity in targeting specific compounds and matrices, shorter processing times, process scalability, and environmental friendliness with no need for harsh solvents [19,312,313]. Additionally, extracts and products attained by EAE have been shown to have heightened biological activities, a probable consequence of a better preservation of the structural integrity of targeted compounds in comparison to other techniques [19,247,314,315]. However, there are also hurdles to the expansion and industry-wide utilization of EAE, such as the lack of stability and loss of the enzymes, interfering or inhibitory factors, and the scarce number of industrially available enzymes that are selective toward substrates only found in seaweed biomass—this has led to the utilization of enzymes more specific to terrestrial feedstocks [19]. This latter fact may lead to slow enzyme kinetics and low substrate specificity. It can also happen that there are no industrial enzymes available to specific substrates only found in seaweed biomass [19]. Equally, this means that discovering and producing novel enzymes from marine sources with high specificity to molecules present in seaweed may greatly strengthen the potential of EAE in terms of enhanced extraction efficiency.

##### Fermentation of Seaweed

Fermentation may also be useful in the extraction and further processing of seaweed biomass [126,141]. Together with hydrothermal treatments, fermentation is also advantageous in that it does not require drying and allows for the utilization of wet seaweed, which, in turn, suits a more environmentally friendly WR approach [126,316]. In addition, fermentation is an expedient process for producing bioethanol from seaweed biomass or, more specifically, from carbohydrates in the biomass [126]. Since these carbohydrate molecules are mostly polysaccharides, prior to fermentation, a hydrolysis step—which may be enzymatic as in EAE—for their conversion into monosaccharides is needed, thereby forming a large variety of sugars [317].

Fermentation has also been reported as a way to improve the nutritional value of seaweed and its biological activities, namely by increasing the concentrations of protein, reducing sugars, fatty acids, and phenolics in the fermentation extract [229,318]. In such studies, fermentation was performed using a seaweed aqueous extract or the wet seaweed biomass with added nutrients—namely, glucose and peptone [229]. Hifney et al. (2018) [229] fermented the brown seaweed *Cystoseira trinodis* with different fungi prior to fucoidan and alginate extraction. All tested fungi synthesized fucoidanase and alginate lyase [229], thereby inducing a reduction in the molecular weight of alginate and fucoidan. Furthermore, there were substantial increases in the fucose and sulfate contents of fucoidan and in the mannuronic/guluronic acid ratio of alginate as a consequence of fermentation [229]. Antioxidant activity, namely as measured by Ferric Reducing Antioxidant Power (FRAP), was increased in the fucoidan and alginate modified by fungal fermentation of *C. trinodis*. The positive effects of fungal fermentation in a study on *S. japonica* and *U. pinnatifida* fermented by the red molds *Monascus purpureus* and *Monascus kaoliang* have been corroborated [318]. Indeed, the phenolic levels of *S. japonica* fermented by *M. purpureus* and *M. kaoliang* were the highest, reaching 67–72 mg GAE/g extract [318]. Protein, reducing sugars, and essential fatty acids contents as well as antioxidant and antidiabetic activities were also increased due to fermentation. The success in applying fermentation processing to brown seaweed species may be due to the biochemical features of their biomass. In fact, factors such as chemical structure and molecular weight affect the fermentability of polysaccharide extracts [319]. It was suggested that carragenophyte red seaweed species are less favorable to this type of fermentation than brown seaweed species, ascribing such a difference to the presence of laminarins, xylofucoglycuronans, and/or xylomannans in brown seaweed biomass [319].

In addition, fermentation can be a key step in the route to produce precursors of biopolymers to be used as bioplastics [141]. In fact, these authors used the spent *Ulva* spp. biomass after ulvan extraction as substrate for biological processing, encompassing dark fermentation and aerobic processing of these compounds by *Cupriavidus necator* bacteria. As a final outcome, it was obtained ~1.6 g/L of biomass with 18.2%, w/dw, polyhydroxybutyrate (PHB), a biopolymer belonging to the polyester group and a biodegradable plastic [141]. Precisely, this experimental work showed the possibility of preparing packaging films using green seaweed biomass as a sustainable feedstock and supplier of ulvan and PHB. The fermentation to generate LA, a biopolymer precursor, is another possibility [320]. These researchers examined the feasibility of producing LA from *U. fasciata*, *G. corticata*, and *K. alvarezii* using *Lactobacillus plantarum* and concluded that the red seaweed *K. alvarezii* supplied a better substrate for the production of LA. Precisely, this red seaweed was the subject of a review by Tabacof et al. (2024) [321], and the utilization of its saccharide fraction for fermentative processes was highlighted. Tabacof et al. (2024) [321] proposed a fermentation biorefinery for *K. alvarezii*, which, after washing, drying, and grinding of the biomass, separated a stream subjected to dilute-acid hydrolysis for the organic phase extraction of HMF and another part, rich in cellulosic material, that was subjected to enzymatic hydrolysis and whose resulting sugars were fermented to produce LA and possibly other molecules. Regarding this subject, Nagarajan et al. (2022) [230] compared green, red, and brown seaweed (*Ulva* sp., *Gracilaria* sp., and *Sargassum cristaefolium*) as potential feedstocks for LA fermentation. After a relatively mild acid and thermal hydrolysis with less than 5% of H_2_SO_4_, *Gracilaria* sp. yielded the highest levels in reducing sugars (0.39 g/g dw), which were fermented to LA [230]. Remarkably, a successful fermentation of the fucose-rich hydrolysate of *S. cristaefolium* to LA was observed [230]. Contrastingly, *Ulva* sp. displayed the worst yields in reducing sugars and overall LA. Nagarajan et al. (2022) [230] also showed that *Lactobacillus rhamnosus* and *L. plantarum* were able to use seaweed sugars as substrate for LA generation.

From this overview, it should be highlighted that, similarly to other presented innovative techniques, fermentation has advantages and shortcomings. It is generally less harmful to the environment, also involving milder conditions that allow for the preservation of sensitive bioactive molecules, and it is a well-known technology already industrialized in other sectors and for other raw materials [126]. Due to low lignin ratios in seaweed, especially if compared to terrestrial plants, structural interference in cellulose extraction by lignin is a secondary issue in applying fermentation to seaweed biomass [322]. Nevertheless, there are substantial problems, such as insufficient conversion ratios of monosaccharides to ethanol, because fermenting organisms have difficulty in fermenting non-glucose sugars that are quantitatively important building blocks of the seaweed polysaccharides. In any case, fermentation as a technological solution for the treatment of seaweed biomass still needs optimization [126].

#### 3.2.5. Challenges in Novel Extractive Techniques: ‘Green’ Solvents, Optimization, Combination, and Biorefinery

All these innovative extraction technologies offer an opportunity and hold potential for future developments that may change the industry, but are still restrained in their use by various problems that may be considered challenges for technologists. Generally, the novel techniques must prove to be technologically viable, environmentally sustainable and non-pollutant, safe, and commercially exploitable, while applicable to the particular components and matricial properties of seaweed biomass. Specifically, this means that all environmental impacts have to be accounted for and minimized—advancing toward a concept of ‘green’ technologies—, safety to final consumers must be ensured and health risks avoided, operational conditions and parameters need to be optimized—maximizing yields and ensuring high selectivity whenever necessary—, and, for a maximal valorization of all components of the biomass, several extraction and separation processes have to be strung together and intelligently articulated in a biorefinery approach.

For environmental friendliness and safety, the disseminated use of ‘green’ solvents may be decisive. Indeed, the coupling of innovative technological processes with the use of ‘green’ solvents is viewed as an alternative way to recover natural substances from biological matrices, thereby preventing the generation of any toxic effluent [200]. There are already various instances where such an alliance between a non-conventional technique and an innovative ‘green’ solvent has been tested. This is the case of the combination of a ‘green’ solvent—choline chloride:1,4-butanediol in a mole ratio 1:5, a DES—and MAE in *F. vesiculosus* by Shang et al. (2021) [257]. Such DES are advantageous with respect to conventional solvents in that they have the ability to develop a wide array of different chemical interactions, ranging from van der Waals interactions to hydrogen bonds and electrostatic attractions. The optimum extraction conditions for maximal extraction yields of polysaccharides allowed a recovery of 116 mg polysaccharides per g of dried seaweed [257]. Moreover, the purified polysaccharides exhibited in vitro antioxidant and anticancer activities [257]. Novel extractive technologies [323,324] can be seamlessly combined with such solvents, including water, for higher yields. Pressurized Liquid Extraction (PLE) is such an example. PLE-based methodologies have been effectively applied to the extraction of polyphenols, especially those more polar. In general, PLE enables improved recovery of natural bioactives when compared to classical extraction techniques [323]. However, there are exceptions. For instance, the ability of SLE and PLE has been compared under several defined conditions to attain antioxidant extracts from different seaweeds (*F. serratus*, *L. digitata*, *G. gracilis*, and *Codium fragile*) [83]. Their results showed that SLE extracts had greater antioxidant capacity than their PLE counterparts [83]. These authors also showed that SLE with cold water had the highest total phenolic content in the case of *F. serratus*, ~81 mg GAE/g, w/dw, vs. ~61 mg GAE/g, w/dw, for the comparable PLE extract of the same seaweed. Another relevant and recent case concerns UAE and NADESs, choline chloride combined with LA [325]. In this study, phenolics and other biologically active (antidiabetic, antioxidant, and anti-hypertensive) compounds were extracted from the red seaweed *Hypnea flagelliformis*. The highest extraction efficiency was achieved with a solvent/solid ratio of ~29:1 and an UAE time of 30 min [325]. In comparison to conventional solvents, including 80%, *v*/*v*, methanol, phenolic levels and antioxidant activity—measured by ABTS, DPPH, and FRAP—of the UAE–NADESs extract were higher [325]. Finally, it should be remarked that EAE, by using water or aqueous solutions under mild conditions of pH and temperature (for optimal enzymatic activity), not only admits and requires ‘green’ solvents, but also ensures chemical safety of the derived extracts.

Besides articulating novel techniques and ‘green’ solvents, such innovative technologies can be brought together in the sense of attaining positive synergies and enhance yields and economic viability. As a representative example, it can be mentioned that a combination of non-thermal HHP technique and EAE—resorting to polysaccharidases—was trialed as an innovative way to increase the extraction of targeted compounds from red seaweed species, *P. palmata* and *S. chordalis* [280]. Biomass was hydrolyzed with cellulase and hemicellulase under HHP (4000 bar applied for 20 min). Subsequently, an improvement in the extraction of protein, polyphenols, and polysaccharides was observed [280]. However, outcomes were claimed to be highly dependent on the seaweed species [280]. Whereas, for *S. chordalis*, antioxidant activity was strongly correlated with polysaccharide and protein contents, for *P. palmata*, correlations were stronger with polyphenol content. Overall, such trials demonstrated the potential of a tandem HHP-EAE in the extraction of relevant phytochemicals from red seaweed. Combining EAE and PEF techniques may also be useful and worth testing [158]. In particular, cellulase addition and PEF together doubled the protein yield from *Ulva* sp. in comparison to opting for only EAE or PEF extractions [326]. However, whenever EAE is considered, the enzyme amount must be weighed against the attained extraction improvement, especially considering that a high enzyme/substrate proportion encumbers any upscaling [307]. Moreover, the combination of UAE with other technologies has been deemed to be advantageous [184]. In particular, it has been leveraged in conjunction with conventional methodologies—comprising maceration, maceration with liquid nitrogen or freezing–thawing technologies—in the extraction of hydrophilic pigment-protein complexes, such as R-phycoerythrin and R-phycocyanin, from red seaweed *Gelidium pusillum* [327]. In the extraction of these compounds, there was evidence supporting a synergistic interaction of UAE and these techniques [184,327].

Going beyond specific tandems of novel techniques (or even novel and conventional techniques) or combinations with ‘green’ solvents, the WR (without drying) approach—an overarching strategy in integrating different operational steps and, necessarily, innovative technology and solvents—may also be an answer to the aforementioned challenges. A WR approach was used for extracting R-phycoerythrin through UAE and EAE from the red seaweed *Grateloupia turuturu* by Le Guillard et al. (2023) [328]. For this purpose, four industrial carbohydrase preparations were combined in accordance with the similarity of their pH and temperature optima and complementarity [329]. Considering that temperature had a negative effect upon the extraction yield, optimized conditions (20 °C for 3 h) were established that enabled an R-phycoerythrin yield of 4.3 mg/g dw, which was 2.3 times higher than the conventional phosphate buffer extraction from dried *G. turuturu* [328]. This study also showed that increased release of R-phycoerythrin and carbohydrates could be linked to the degradation of constitutive polysaccharides in *G. turuturu*, resulting in a reduction in the average molecular weight by a factor of 2.2. Hence, it has been demonstrated that an optimized UAE–EAE within a WR approach is a viable and efficient strategy in R-phycoerythrin extraction from *G. turuturu*, thereby avoiding expensive pre-treatment—namely, biomass drying—as in the conventional approach [328]. Das et al. (2025) [330] carried out research into UAE and MAE—both applied individually and simultaneously (UAE–MAE)—for extracting bioactive compounds from *A. esculenta*. Just as Le Guillard et al. (2023) [328], Das et al. (2025) [330] adopted the WR approach. Moreover, the latter evaluated microwave (336–1340 W) and ultrasound (50–200 W) power as well as operational time (5–20 min), choosing water as a ‘green’ solvent. Das et al. (2025) [330] reported that while the highest soluble carbohydrate levels (~33 mg glucose equivalent/100 mg extract) were reached with UAE (using 200 W for 20 min), UAE–MAE (using 50 W ultrasound and 1340 W microwave power for 10 min) led to the highest phenolic contents (>2 mg GAE/100 mg extract) and relevant antioxidant activity. In addition, the efficacy of the three extraction alternatives was assessed by scanning electron microscopy, which showed clear cell damage by all techniques [330].

This progressive integration of different novel techniques and solvents in more environmentally sustainable approaches is conducive to the development of a full-fledged and effective biorefinery taking seaweed biomass as an excellent feedstock. Precisely, regarding the conjugation of novel techniques in a biorefinery approach, Herrera Barragán et al. (2022) [331] addressed the feasibility of an alternative multi-product biorefinery scenario based on ‘green’ technologies in the case of *S. latissima*. As paradigmatic of an emerging sustainable technology targeting alginate, fucoidan, and laminarin in *S. latissima*, EAE has been identified and selected [331]. The EAE-based biorefinery was compared to an alternative that employs an alkaline extraction technique currently used in the industry. The latter was characterized by low yields and was economically unviable in Europe [331]. On the other hand, EAE-based biorefinery was advantageous in that it achieved full biomass valorization by processing by-products into complementary products in the fields of agriculture and husbandry. Ceaser et al. (2025) [41] presented a seaweed biorefinery concept encompassing nanocellulose production. This involved Soxhlet extraction of lipids and lipophilic components from the seaweed as the first step, followed by UAE combined with a DES for releasing alginate, hemicellulose, and protein, thus leaving a spent biomass that may be subjected to bleaching with hydrogen peroxide in order to attain purified cellulose fiber. This material can then be submitted to additional treatments—using enzymatic, chemical, mechanical, and ultrasonication means—for production of bioethanol together with cellulose nanocrystals and nanofibrils, which find applications across composite materials, fiber, and medical industries [41]. Another case of biorefinery comprising innovative techniques was advanced by Manikandan and Lens (2023) [141], whose study embedded a dark fermentation and an aerobic fermentation for polyhydroxyalkanoates (biopolymers) in a wider sequence of operational processes for extracting all potentially valuable components from *Ulva* sp. Another recent study [155] went further afield and used HydroDynamic Cavitation (HDC)—a scalable operation equivalent to ultrasound cavitation that has emerged as a potential extraction tool and an almost untested technique in seaweed—to build a biorefinery process for brown seaweed using *A. esculenta* as a representative species of this taxonomic group. Processing time, solvent, and HDC operational parameters were fine-tuned in order to extract alginate, laminarin, mannitol, and protein in a cascading manner [155]. Membrane ultrafiltration was also employed as a technology to separate laminarin and mannitol. As evidence of the proposed biorefinery’s prowess, it may be noted that the purity of the obtained laminarin—displaying biological activities identical to those of commercially available products—was nearly 87% and the recovery rate reached ~56% of the initially present content in the seaweed [155]. This is improvable, but these are already remarkable results for such a novel approach and poorly studied process like HDC [155]. This experimental work also showed that an HDC-assisted biorefinery can reduce energy consumption and, as such, carbon footprint.

The biorefinery concept can also be extended to encompass the cultivation of seaweed as well as its harvest and processing, taking into account innovative sea farming techniques, such as Integrated Multi-Trophic Aquaculture (IMTA), that are less resource demanding and environmentally friendlier [332]. Moreover, cultivation opens new venues for the control and modulation of the seaweed biomass composition, thus tailoring to some extent its contents to an optimal biorefinery that valorizes all components. Namely, green seaweed species, such as *Ulva ohnoi*, can be rich sources of starch depending on cultivation conditions and seasonality [333]. Indeed, nutrient starvation was shown to augment starch content up to 21.4%, w/dw, thereby modulating this biomass to fit a biorefinery process built around starch extraction as its main vector. A biorefinery was proposed, conjugating starch recovery after cell disintegration with lipid, protein, and ulvan extraction and possibly allowing for the application of innovative chemical and enzymatic means to separate and refine particular fractions [333].

Accordingly, there is a wide field of possibilities opening up with the conjugation of a biorefinery philosophy with cutting-edge technologies. Namely, with respect to novel cell disruption techniques, Saravana et al. (2023) [334] list a series of possible technological routes, ranging from application of SLE with surfactant or switchable solvents, DES, NADES, or other IL, to explosive decompression and compressional-puffing, and also including high voltage electrical discharge, ozonation, or plasma utilization. All these technological solutions require further study that may confirm whether they will be effective in the recovery of value-added compounds from seaweed while upholding the principles of ‘green’ chemistry and sustainability [334]. These technologies may be fitted into larger flow diagrams and strategies in a way that maximizes efficacy gains and spares resources. Indeed, synergies between upstream and downstream technologies have to be explored in order to ensure the commercial viability of seaweed biorefinery systems, being also specifically tailored to local specificities and available resources [334].

For all proposed innovative technologies, a proper and thorough assessment of the biological activity and molecular structures of the recovered components must be performed in order to ensure that there are no hidden problems of quality losses [200]. All these novel technological solutions have disadvantages and application hurdles, such as high capital investment and operational costs, upscaling difficulties in the transition to an industrial level of operation—a transition that is easier whenever the operation is continuous [335]—, overly contrived configurations in many instances, and technological immaturity or insufficient readiness level. Thus, further research focused on comparing alternative methods and the performance of cost/benefit analyses has critical importance [200]. This has to be coupled with a continuous striving for improvements in the extraction processes in order to find optimal levels for the various operational parameters—including RSM and similar experimental design approaches—and a combination of different techniques may require optimization for a successful implementation of a biorefinery approach [25,30,80,197,200,236,257,267,279,328]. Additionally, such efforts at optimizing processes may benefit from recent advances in bioinformatics, with flux balance analysis being used as a computational tool to foresee results/outcomes prior to carrying out any experiment involving the processes [138,220]. Mathematical algorithms, statistics, and adequately extensive databases are necessary for building predictive models that accurately simulate the phenomena underlying extraction processes. Hence, though promising, bioinformatics depends also on further experimental work, especially considering data scarcity in many core aspects and regarding novel techniques.

## 4. Conclusions and Future Challenges

This scoping review of the literature showed that the growing importance of seaweed biomass in terms of acknowledgment and utilization of its still largely untapped biotechnological potential (invaluable compounds with multiple biological activities), as well as in terms of economic impact (including expansion of seaweed farming), has led to a spurt of basic and applied research in phycology during the last decade. Regarding applied research, this reality has fostered studies on the extraction of nutrients and bioactive compounds from seaweed biomass, thereby covering not only amelioration and testing in new species of conventional extracting techniques, but above all, the experimental conception and development of innovative and cutting-edge technologies applied to the seaweed matrix. This has been coupled with novel approaches and strategies in fully exploiting the biotechnological potential lying within seaweed biomass through an intelligent articulation of processing operations and their optimization. These new techniques and approaches are much needed, given the technological difficulties in disrupting seaweed cell walls and releasing all relevant intracellular components, preserving all the biological activity of the most sensitive and labile molecules, protecting the environment (including lessening the carbon footprint), and ensuring high standards of quality and safety for the attained final products. If the novel methodologies and approaches succeed in mastering these difficulties and advance to a safer and ‘greener’ processing of the whole seaweed biomass—thus building biorefineries with seaweed as feedstock—, then the sector of seaweed-derived products may become a fundamental pillar of a XXI^st^ Century bridge into the future by supporting the transition to a blue economy.

For such a transition to occur, it is crucial to ensure that new technologies are harmoniously welded into biorefinery concepts in an economically feasible way, which presupposes that all devised operational steps—including cell wall disruption techniques—must be up-scalable to an industrial level. Adjustment and fine-tuning of the operational conditions and parameters must always be performed at this larger scale, but it also profits from models that draw on the results of previous optimization studies at a laboratory- or pilot-scale. In this upscaling challenge, developing batch into continuous processes is crucial and often entails a fundamental rethinking of conditions and whole process routes. All mentioned obstacles are compounded by the sheer taxonomic, biochemical, and cell structure variety of seaweeds, which makes it very difficult to find and optimize a universal extracting technique that can be applied to the commercial processing of any species’ biomass. As shown in this review, there are studies that attempted to test technological innovations across species, but were necessarily limited to a few representative species of the major brown, green, and red groupings. The evaluation of their overall efficiency or extraction yields, as well as the quality and biological activity of the extracts, often revealed large differences in outcomes as a function not only of the major grouping to which the seaweed belongs, but also of its particular species—even taxonomically near species subjected to the same processes had diverging results. Hence, despite the positive results of some techniques, any universally viable technique is a chimera and neither technologies based on physical mechanisms, such as MAE or UAE, nor on enzymatic selectivity, i.e., EAE, are able per se to master this challenge.

The application of ‘green’ solvents, regardless of being DES, NADES, water, ethanol, or any other, is also variably effective, depending on matrix and the nature of the extracting technique—in some instances, such as SWE, only a particular solvent or a narrow list of solvents is possible. This makes fitting different techniques, conditions, and extracting media together into a seamless processing route a necessity and a further challenge. This route may be DR, WR, or any other novel concept. However, it will only succeed if all its operational steps and final outcome meet the requisites of technological viability, commercial feasibility, environmental friendliness, and safety. For this reason, besides yield calculations and extract characterizations, complete environmental sustainability and economic studies, including LCA, are essential.

In any case, recent research is making inroads into increasing yields, preserving purity, and protecting the environment and consumer health by applying entirely novel sets of technologies and solvents, exploring previously poorly studied species, optimizing parameters with RSM and more advanced modeling, and bringing all together with smartly designed processing strategies. Therefore, past progress, especially in recent years, signals that a renewed ‘bluer’ and ‘greener’ seaweed processing industry, a new generation of seaweed-derived products, and a much vaster utilization and valorization of this major marine resource are within reach.

## Figures and Tables

**Table 1:** Overview of scientific studies concerning conventional extraction techniques and approaches applied to seaweed biomass.

Approach/Technique	Operational Conditions	Study Outcome	Reference
DR/SLE	Brown seaweed *N. zanardinii* was extracted with water using a 1:20 biomass/solvent ratio and stirring for 6 h at 65 °C	Fucoidan extraction yield was 5.2%	[78]
	Brown seaweed *A. nodosum* was extracted with ethanol/water (8:2, *v*/*v*) three times for 36 h and then with hot water	Polysaccharide extraction yield of 9.3% when extracted with hot water at 83.9 °C for 4.3 h and a dry biomass/water ratio of 1:26.9 (*w*/*v*)	[79]
	Brown seaweed *S. vestitum* was extracted with ethanol/water (7:3, *v*/*v*) at 30 °C for 12 h using 1:50 (*w*/*v*) biomass/solvent ratio	Total polyphenol extraction was 40.3 mg GAE/g dw	[80]
	Brown seaweed *S. japonica* was extracted with either water, ethanol, acetone, dichloromethane or diethyl ether using a 1:32 biomass/solvent ratio, 24 h extraction time, and 500 rpm stirring velocity at room temperature	Phenolic extraction yield was optimal with water, reaching 2.4 mg PhloroGlucinol Equivalent (PGE)/g dw; other conventional solvents did not surpass 0.6 mg PGE/g dw	[81]
	13 species (4 brown, 8 red, and 1 green) extracted with hexane, CH_2_Cl_2_, ethyl acetate, methanol, and water in a 1:30 (*w*/*v*) ratio; for all solvents except water: triple extraction at room temperature for 24 h each; for water: triple extraction at 80 °C for 3 h each	Aqueous extraction led to better yields: from 9.5% in *D. polypodioides* to 58.5% in *Gracilaria domingensis*	[82]
	4 seaweed species extracted with methanol/water (7:3, *v*/*v*), ethanol/water (8:2, *v*/*v*), cold water, and hot water in a 1:20 (*w*/*v*) ratio for pure aqueous and 1:10 (*w*/*v*) in the other extractions and at room temperature for 24 h, with exception of hot water (60 °C for 24 h)	Cold water presented the highest extraction yields, ranging from 35.9% in *F. serratus* to 39.5% in *L. digitata* and 48.2% in *C. fragile*, with exception of *G. gracilis*, whose highest yield was attained with methanol/water (7:3, *v*/*v*), 29.2%	[83]
	Brown seaweed species *A. nodosum* and *L. hyperborea* were extracted with either water or 0.1 M HCl at 70 °C for 2.5 h using a 1:20 (*w*/*v*) biomass/solvent ratio	Laminarin content in the extracts was highest with water than with 0.1 M HCl, 4.6 vs. 4.3% in *A. nodosum* and 4.4 vs. 3.2% in *L. hyperborea*	[84]
	2 green seaweed (*C. tomentosum* and *U. lactuca*), 4 red seaweed (*C. crispus*, *G. gracilis*, *P. palmata*, and *P. dioica*), and 4 brown seaweed species (*E. bicyclis*, *F. vesiculosus*, *H. elongata*, and *U. pinnatifida*) were extracted by SLE and a BBD/RSM was applied for parameter optimization: time (1, 3, 5 h), temperature (25, 50, 75 °C), and biomass/water ratio (1:25, 1:50, 1:75, *w*/*v*)	The highest ABTS in relation to the Antioxidant Potency Composite Index (APCI) was attained for *P. palmata*, 10.1% vs. 9.1%, respectively, with SLE at 25 °C for 3 h and a biomass/water ratio of 1:75, *w*/*v*, but still lower than the SWE technique, 12.2–14.4%	[85]
	9 brown seaweed species extracted with hexane, chloroform, ethyl acetate, acetone, and ethanol in a 1:33 (*w*/*v*) ratio at 50 °C for 24 h using 150 rpm stirring	Extraction yield increased partially with polarity, and ethanol enabled the highest yields, ranging from 14.6% in *F. spiralis* to 24.1% in *Bifurcaria bifurcata*, 27.0% in *H. elongata*, and 38.8% in *U. pinnatifida*	[86]
	Brown seaweed *Padina australis* was subjected to cold maceration SLE at room temperature for 24 h (repeated three times) using a 1:2 (*w*/*v*) biomass/ethanol ratio	SLE extracts contained alkaloids, flavonoids, steroids, saponins, and tannins, exhibiting antibacterial activity	[87]
	Hexane, ethanol, and acetone:methanol (1:1, *v*/*v*) were used on *S. japonica* and *Sargassum horneri* in a 1:20 (*w*/*v*) ratio at 25 °C for 20 h with 300 rpm stirring	Oil extraction yields: 1.2% in *S. japonica* and 1.3–1.4% in *S. horneri*	[88]
	Brown seaweed *L. japonica* was subjected to acid, water, and alkali extraction at 80 °C and twice using a 1:50 (*w*/*v*) ratio; acid (1%, *w*/*v*, citric acid, pH 2.0) and alkali (1%, *w*/*v*, NaOH, pH 10.0) extractions took 4 h, and water extraction only 2 h	Polysaccharide extraction yield varied from 10.3% with water to 44.6% in the case of alkali extraction	[89]
	4 brown seaweed species (*Sargassum aquifolium*, *S. cristaefolium*, *Sargassum polycystum*, *T. ornata*) were subjected to cold maceration SLE at room temperature for 24 h (repeated three times) using a 1:10 (*w*/*v*) biomass/ethanol ratio	Extraction yield and total flavonoid contents were 2–3% and 400–600 mg quercetin/g dw, respectively	[90]
	Red seaweed *S. chordalis* and brown seaweed *S. muticum* were extracted with chloroform:methanol (1:1, *v*/*v*) in a 1:3 (*w*/*v*) biomass/solvent ratio at room temperature and for 12 h (repeated three times); *S. muticum* was also extracted with chloroform in a 1:5 (*w*/*v*) biomass/solvent ratio at room temperature and for 12 h	The highest lipid recovery was achieved in *S. muticum* with chloroform:methanol (1:1, *v*/*v*), 3.2% lipid yield; for the same conditions, lipid yield was 3.0% in *S. chordalis*	[91]
	11 brown seaweed species (*A. esculenta*, *A. nodosum*, *F. serratus*, *F. spiralis*, *F. vesiculosus*, *Halidrys siliquosa*, *H. elongata*, *L. digitata*, *L. hyperborea*, *L. saccharina*, and *Pelvetia caniculata*) were extracted with ethanol/water (5:5, *v*/*v*) using a biomass/solvent ratio of 1:15, *w*/*v*, at 20 °C for 4 h	Extraction yield ranged between 10.5% in *F. serratus* and 19.3% in *L. hyperborea*	[92]
	*S. latissima* dry biomass:anhydrous sodium sulfate:ethyl acetate in 4:11:20 (*w*/*w*/*v*) ratio, 60 min at 1373 rpm shaking	Relative lipid yield from *S. latissima*: 27.3%	[73]
DR/Soxhlet	Brown seaweed *Colpomenia sinuosa* was extracted alternatively with cyclohexane, CH_2_Cl_2_:methanol (1:1, *v*/*v*), methanol, and water in a 1:10 (*w*/*v*) biomass/solvent ratio for 72 h at room temperature; Soxhlet extractions were performed with the same 4 solvents for 6 h	The most potent extract was obtained by Soxhlet using CH_2_Cl_2_:methanol (1:1. *v*/*v*) solvent, and it displayed anti-tumoral effects	[93]
	Brown seaweed *Posidonia oceanica* was extracted with ethanol:toluene (1:2, *v*/*v*) in a 1:22 (*w*/*v*) biomass/solvent ratio for 6 h in a Soxhlet system	Polysaccharide (cellulose) extraction yield was 32.5% with ethanol:toluene (1:2, *v*/*v*)	[94]

**Table 2:** Overview of scientific studies concerning innovative extraction techniques based on physical, chemical, and biological processes and applied to seaweed biomass.

Approach/Technique	Operational Conditions	Study Outcome	Reference
DR/EAE	Brown seaweed *N. zanardinii* was extracted by EAE using four alternative enzymatic treatments: (i) Alcalase (5%, *v*/*v*), pH 8.0 at 50 °C for 24 h; (ii) Celluclast (5%, *w*/*v*), pH 4.5 at 50 °C for 24 h; (iii) Viscozyme (5%, *v*/*v*), pH 4.5 at 50 °C for 24 h; and (iv) Flavourzyme (5%, *v*/*v*), pH 7.0 at 50 °C for 24 h	Fucoidan extraction yield varied from 4.3% with Viscozyme treatment to 4.8% with Celluclast and to 5.6% with Alcalase and encompassing 4.4% with Flavourzyme treatment	[78]
	Green seaweed *U. fenestrata* was extracted by EAE using a 1:20, *w*/*v*, biomass/solvent ratio; two cellulase blends, Viscozyme L and Cellulysin, in 0.1 M sodium acetate buffer, and two proteases, Neutrase 0.8 L and Flavourzyme, in 0.1 M Tris HCl buffer, were tested; Viscozyme L was tested at pH 5 and 50 °C, Cellulysis at pH 5 and 40 °C, Neutrase 0.8 L at pH 7 and 60 °C, and Flavourzyme at pH 5 and 50 °C; four different extraction times were assayed (3, 6, 17, and 20 h)	Ulvan extraction yield reached a maximum of ~14%, w/dw, with Cellulysis, after 20 h of cellulase treatment; the comparable yield for Neutrase 0.8 L was ~13%, w/dw, and for Viscozyme L and Flavourzyme was ~12%, w/dw; higher extraction time led to higher yields, thereby ranging from 3 to 6%, w/dw, after 3 h, to 12–14%, w/dw, after 20 h	[228]
	Brown (*F. vesiculosus*), green (*U. rigida*), and red (*G. vermiculophylla* and *P. dioica*) seaweed species were extracted by EAE; parameter optimization was carried out for *G. vermiculophylla* applying sequentially cellulolytic (Viscozyme at pH 4.5) and proteolytic enzymes (Flavourzyme and papain at pH 7.0) at 50 °C; firstly, cellulolytic enzyme concentration (3.1–61.9 U/g seaweed), biomass/solvent ratio (1:9 to 1:100, *w*/*v*), and time (7.9–28.1 h) were optimized; and secondly, optimal proteolytic enzyme concentration (1378.7–5621.3 U/g seaweed) and time (1.2–6.8 h) were established	For *G. vermiculophylla*, while cellulolytic yield varied from 17.6% with the lowest Viscozyme level (3.1 U/g), 1:17, *w*/*v*, biomass/solvent ratio, and 18 h to 42.4% with 50 U/g, 1:33, *w*/*v*, biomass/solvent ratio, and 24 h, proteolytic yield ranged from 36.9 to 38.2% with 3500 U/g and only 1.2 h to 48.9–51.8% with 5621 U/g and 4 h (the lower end of the intervals results from Flavourzyme and the upper end from papain); sequential EAE improved overall extraction yield by 30–160% in comparison to conventional SLE; for all tested species with exception of *F. vesiculosus*, yield was enhanced by adding a proteolytic stage to the cellulolytic one; for all seaweed species, yield was increased with a cellulolytic treatment prior to proteolysis	[217]
DR/Fermentation	Brown seaweed *C. trinodis* was subjected to a semi-solid fermentation for fucoidan and alginate extraction purposes; both seaweed biomass and water were sterilized by autoclaving at 121 °C for 20 min; fungal inocula of *Aspergillus niger*, *Chaetomium funicola*, *Dendryphiella arenaria*, *Emericella nidulans*, *Eurotium chevalieri*, and *Stachybotrys chartarum* were attained from 3 seaweed species; 1:20, *w*/*v*, biomass/water ratio was used and 1%, *v*/*v*, inoculum was added to the biomass–water mixture, being fermentation carried out at 28 °C and with continuous shaking (120 rpm) for 3 days	Fucoidan extraction yield was higher with the fungal inoculum from *C. funicola*, 4.4%, than with all other inocula, 3.4–3.9%; alginate extraction yield was higher with the fungal inoculum from *E. chevalieri*, 21.8%, than with all other inocula, 17.4–18.9%	[229]
	Green (*Ulva* sp.), red (*Gracilaria* sp.), and brown (*S. cristaefolium*) seaweed species were studied as feedstocks for LA fermentation; previous acid thermal hydrolysis (<5% sulfuric acid, 121 °C, 20 min); fermentation with *Lactobacillus* sp., and *Weissella* sp.	Maximum reducing sugar recovery, 0.39 g/g seaweed, and LA yield, 0.94 g/g, was achieved with *Gracilaria* sp.; for *S. cristaefolium*, 0.36 g/g seaweed in reducing sugar and 0.81 g/g LA yield; for *Ulva* sp., 0.21 g/g seaweed in reducing sugar and 0.85 g/g LA yield (note however the very low sugar recovery for LA production)	[230]
DR/MAE	Brown seaweed *S. vestitum* was extracted with ethanol/water using 1:50 (*w*/*v*) biomass/solvent ratio and MAE frequency of 2450 MHz; a BBD in an RSM was applied for parameter optimization: MAE power (60, 80, 100% of 1200 W), ethanol/water proportion (3:7, 5:5, 7:3, *v*/*v*), and time (25, 50, 75 s)	Best total polyphenol extraction (58.2 mg GAE/g dw) and more favorable antioxidant properties were attained with the following optimal MAE conditions: 960 W power (80% of 1200 W), 7:3, *v*/*v*, ethanol/water, and 75 s irradiation	[80]
	Brown seaweed *A. nodosum* was extracted by either MAE or UAE-MAE with 0.1 M HCl, using a biomass/solvent ratio of 1:10, *w*/*v*, at 37.7–92 °C in MAE and 36.2–98 °C in UAE-MAE, for 2 or 5 min, and applying variable microwave power (250, 600, 1000 W) at 2450 MHz	Applying 1000 W microwave power for 5 min increased total soluble carbohydrate yield from 0.5 to 1.5 to 3.2 g glucose equivalent/100 g dw; maximum yields were attained with UMAE: total soluble carbohydrates (10.4 g glucose equivalent/100 g dw), fucose-sulfated polysaccharides (3.5 g fucose/100 g dw), and phenolic compounds (2.6 GAE/100 g dw)	[231]
	Brown seaweed *Sargassum swartzii* was extracted with ethanol/water; an RSM was applied for parameter optimization: MAE power (174, 240, 400, 560, 626 W), biomass/solvent ratio (1:23, 1:25, 1:30, 1:35, 1:37, *w*/*v*), ethanol/water percentage (31.7, 40.0, 60.0, 80.0, 88.3%, *v*/*v*), and time (23.8, 30, 45, 60, 66.2 min)	Optimal phlorotannin recovery of 5.59 mg Phlorotannin Equivalent/g dw was achieved with 613 W MAE power, 1:33, *w*/*v*, biomass/solvent ratio, 52.0%, *v*/*v*, ethanol/water percentage, and 65 min	[232]
	Brown seaweed *N. zanardinii* was extracted with HCl aqueous solution; a BBD in an RSM was used for parameter optimization: temperature (45, 60, 75 °C), time (10, 20, 30 min), MAE power (300, 400, 500 W), and biomass/solvent ratio (1:10, 1:20, 1:30, *w*/*v*)	Alginate extraction yield was 31.4% for the optimized conditions: 67 °C, 19 min, 400 W, and 1:29, *w*/*v*, biomass/solvent ratio	[233]
	Green seaweed species (*Ulva* spp. and *Monostroma latissimum*) were extracted with water, and the effect of temperature (100, 120, 140, 160, 180 °C) on yields and properties was studied; a 1:20 biomass/water ratio was combined with a maximal microwave power of 1000 W at 2450 MHz for 10 min	Solubilization rate increased from ~30–50% to ~70–90% when temperature increased from 100 to 180 °C; while ulvan yields were 37–40% from *Ulva* spp. subjected to MAE at 160 °C, rhamnan sulfate yield was 53% from *M. latissimum* at 140 °C	[234]
	Brown seaweed *A. nodosum* was extracted with aqueous acid solutions and a MAE parameter optimization was carried out: acid concentration (0.01, 0.05, 0.1, 0.2, 0.4 M H_2_SO_4_), temperature (120, 150, 180 °C), biomass/solvent ratio (1:18, 1:32, 1:159, *w*/*v*), and time (1, 5, 10, 20, 30 min)	Monosaccharide extraction yield of 12.7% was achieved with 0.4 M H_2_SO_4_, 150 °C, 1:32 biomass/solvent ratio, and 1 min MAE treatment; monosaccharide extraction yield increased with H_2_SO_4_ concentration and it was lower at 120 and 180 °C	[235]
	Green seaweed *U. prolifera* was extracted with aqueous acid solutions, and a MAE parameter optimization was performed for acid concentration (0.01, 0.05, 0.1 M HCl) and temperature (90, 120, 150 °C), while 1:20, *w*/*v*, biomass/solvent ratio, microwave power of 500 W at 2450 MHz, and 15 min time were constant	Total solubilization rate reached ~70% for 0.1 M HCl MAE at 150 °C, thereby increasing with acid concentration and temperature; polysaccharide yield was ~35% for 0.01 M HCl MAE at 120 °C, decreasing with acid concentration and temperatures higher than 120 °C; however, polysaccharides extracted with 0.1 M HCl MAE and 150 °C displayed higher antioxidant activity	[236]
	3 brown seaweed species (*F. vesiculosus*, *F. spiralis*, and *L. saccharina*) were extracted by MAE with 0.1 M HCl aqueous solution (containing 2 M calcium chloride, CaCl_2_) and parameter optimization was carried out: MAE power (240, 560 W), biomass/solvent ratio (1:10, 1:25, *w*/*v*), and time (60. 120 s)	A 1:25, *w*/*v*, biomass/solvent ratio led to better results, being maximal yield, 12.3%, achieved with 240 W and 60 s, and optimal fucoidan recovery, 0.77 mg/mg extract, with 560 W and 120 s	[237]
DR/PLE	4 seaweed species extracted with methanol/water (7:3, *v*/*v*), ethanol/water (8:2, *v*/*v*), and water; methanol/water PLE was carried out at 90 °C and under 69 bar for 25 min; ethanol/water PLE was carried out at 100 °C and under 69 bar for 25 min; water PLE was carried out at 120 °C and under 104 bar for 25 min	Water PLE presented the highest extraction yields, varying from 26.9% in *G. gracilis* to 33.4% in *F. serratus*	[83]
	Brown seaweed *Nemacystus decipiens* was extracted by PLE with water (biomass/water ratio of 1:15, *w*/*v*) at ~35 °C and under 400, 700 or 1000 bar for one to three consecutive cycles	Fucoidan extraction yield was 16.7% for the best PLE conditions, 700 bar and two consecutive cycles, thereby presenting a value lower than the SLE yield with water, 18.1%; however, PLE fucoidan displayed higher antioxidant activity than SLE fucoidan	[238]
	*H. elongata* extracted at 4 different temperatures (50 °C, 100 °C, 150 °C, 200 °C) for 20 min using hexane, ethanol or water	Extraction yield from *H. elongata* increased with temperature and solvent polarity: 3.4–7.6% (hexane); 8.3–36.9% (ethanol); 9.5–51.6% (water)	[77]
DR/SFE	Supercritical CO_2_ (0.17:1/min, *v*/*w*, flow rate) was used on *U. pinnatifida* at 40 °C and under 400 bar for 3 h	Total extraction yield of 1.2% and recovered fucoxanthin reached nearly 80 mg/g of extract in *U. pinnatifida*, being the latter inversely correlated with temperature	[239]
	Supercritical CO_2_ (0.27:1/min, *w*/*w*, flow rate) and several co-solvents (sunflower oil, soybean oil, canola oil, ethanol, water) were applied to *S. japonica* using three sets of conditions for 4 h: (i) 0.50% co-solvent at 45 °C and under 200 bar; (ii) 1.25% co-solvent at 50 °C and under 250 bar; (iii) 2.00% co-solvent at 55 °C and under 300 bar;	Carotenoid extraction yield: 2.4 mg of total carotenoids per g dw with canola oil and sunflower oil using 2.00% co-solvent at 55 °C and under 300 bar	[240]
	Supercritical CO_2_ (0.27:1/min, *w*/*w*, flow rate) and co-solvent ethanol (0.01:1/min, *v*/*w*, flow rate) were used on *S. japonica* and *Sargassum horneri* at 45 °C and under 250 bar for 2 h	Oil extraction yields: 1.1% in *S. japonica* and 1.3–1.4% in *S. horneri*	[88]
	Supercritical CO_2_ (0.33:1/min, *w*/*w*, flow rate) with and without ethanol as co-solvent was used on *S. chordalis* and supercritical CO_2_ (0.33:1/min, *w*/*w*, flow rate) only was used on *S. muticum*, all performed at 45 °C and under 290 bar	The lipid recovery through supercritical CO_2_ and co-solvent ethanol (2%, *w*/*w*) was 41% of the initial lipid content in *S. chordalis*, but only 25% in *S. muticum*	[91]
	Supercritical CO_2_ and co-solvent ethanol were used on *U. pinnatifida* and a BBD in an RSM was applied for parameter optimization: flow rate (0.2:1, 0.5:1, 0.8:1/min, *v*/*w*), ethanol amount (1.250, 3.125, 5.000 mL), particle size (100, 450, 800 μm), temperature (20, 50, 80 °C), pressure (69, 241, 414 bar), and time (30, 135, 240 min)	Total flavonoid content of 31.76 mg/g and fucoxanthin content of 20.42 mg/g were reached with optimized conditions: 0.8:1/min, *v*/*w*, CO_2_ flow rate, 3.0 mL ethanol, 100 μm particle size, 48 °C, 400 bar, and 230 min	[241]
DR/SWE	Brown seaweed *N. zanardinii* was extracted by SWE, and a BBD/RSM was applied for parameter optimization: time (10, 20, 30 min), temperature (90, 120, 150 °C), and biomass/water ratio (1:20, 1:30, 1:40, *w*/*v*)	Fucoidan extraction yield was 26.0% for the optimized conditions: 29 min, 150 °C, and 1:21, *w*/*v*, biomass/water ratio, which compares to 5.2% yield with conventional DR/SLE applied to the same seaweed species	[242]
	Brown seaweed *E. maxima* was extracted by SWE and a central composite experimental design (RSM) was applied for parameter optimization: time (5, 10, 15, 20, 30 min), temperature (100, 120, 140, 160, 180 °C), and biomass/water ratio (1:10, 1:20, 1:30, 1:40, 1:50, *w*/*v*)	Whereas optimal phenolic extract yield was ~76% for 180 °C, 23.75 min, and water/biomass ratio of 1:30, *w*/*v*, optimal polysaccharide yield was ~58% for 120 °C, 5 min, and water/biomass ratio of 1:30, *w*/*v*	[243]
	Brown seaweed *S. japonica* was extracted by SWE with water or a 0.5 M IL, 1-Butyl-3-MethylImidazolium TetraFluoroBorate (BMITFB), aqueous solution, thereby keeping a 1:32 biomass/solvent ratio, a 50 bar pressure, and a 5 min extraction time and varying temperature (100, 125, 150, 175, 200, 225, 250 °C); alternative BMITFB concentrations in aqueous solution were also tested: 0.25, 0.75, and 1.0 M	Phenolic extraction yield was optimal with 0.25 M BMITFB aqueous solution and 175 °C, reaching ~60 mg PGE/g dw; 0.5 M BMITFB aqueous solution was only better than water at 150 and 250 °C; while, for water, optimal temperature range was 175–200 °C, for 0.5 M BMITFB aqueous solution, optimal temperature was between 150 and 175 °C; higher BMITFB concentration led to lower total phenolic content in the extracts, reaching ~20 mg PGE/g dw for 1 M BMITFB; conventional extractions did not surpass 2.4 mg PGE/g dw	[81]
	Brown seaweed *U. pinnatifida* was extracted by SWE, and different operational parameters were tested: time (5, 10, 15, 20, 30 min), temperature (120, 150, 180, 210 °C), and biomass/water ratio (1:50, 1:100, *w*/*v*)	Fucoidan extraction yield was maximal (46 mg/g dw) with a SWE combination of 5 min, 120 °C, and 1:50, *w*/*v*, biomass/water ratio	[244]
	2 green seaweed (*C. tomentosum* and *U. lactuca*), 4 red seaweed (*C. crispus*, *G. gracilis*, *P. palmata*, and *P. dioica*), and 4 brown seaweed species (*E. bicyclis*, *F. vesiculosus*, *H. elongata*, and *U. pinnatifida*) were extracted by SWE using 140 °C and 20 bar or 190 °C and 30 bar, always for 30 min; biomass/water ratio was 1:75, *w*/*v*, with exception of *U. lactuca* and *U. pinnatifida*, which required 1:100, *w*/*v*	APCI reached a maximal value of 46% for *E. bicyclis*, being the highest ABTS and FRAP attained with SWE at 190 °C and 30 bar applied to *E. bicyclis*, thereby surpassing an optimized SLE and UAE techniques	[85]
	Brown seaweed *S. japonica* was extracted by SWE and a BBD/RSM was used for parameter optimization: time (5, 10, 15 min), temperature (100, 140, 180 °C), pressure (20, 50, 80 bar), agitation speed (100, 200, 300 rpm), and biomass/water ratio (1:11, 1:15, 1:25, *w*/*v*)	Fucoidan extraction yield was 13.6% for the optimized conditions: 12 min, 127 °C, 80 bar, 300 rpm, and 1:21, *w*/*v*, biomass/water ratio	[245]
DR/UAE	Brown seaweed *S. vestitum* was extracted with ethanol/water (7:3, *v*/*v*) at 30 °C for 60 min using a 1:50 (*w*/*v*) biomass/solvent ratio	Total polyphenol extraction was 48.5 mg GAE/g dw	[80]
	Brown seaweed *A. nodosum* was extracted by either UAE or UAE-MAE with 0.1 M HCl, using a biomass/solvent ratio of 1:10, *w*/*v*, at room temperature in UAE and up to 98 °C in UAE-MAE, for 2 or 5 min, and applying variable ultrasonic amplitude (20, 50, 100% of 500 W) and microwave power (250, 600, 1000 W)	Applying 50% ultrasonic amplitude for 5 min increased total soluble carbohydrate yield from 1.5 to 2.0 to 2.5 g glucose equivalent/100 g dw; maximum yields were attained with UMAE: total soluble carbohydrates (10.4 g glucose equivalent/100 g dw), fucose-sulfated polysaccharides (3.5 g fucose/100 g dw), and phenolic compounds (2.6 GAE/100 g dw)	[231]
	Brown seaweed species *A. nodosum* and *L. hyperborea* were extracted with either water or 0.1 M HCl for 15 min using a 1:20 (*w*/*v*) biomass/solvent ratio, 20 kHz frequency, and 35.61 W/cm^2^ ultrasonic intensity	Laminarin content in the extracts was highest with 0.1 M HCl solvent, reaching 5.8% in *A. nodosum* and 6.2% in *L. hyperborea*; SLE did not surpass 5%	[84]
	2 green seaweed (*C. tomentosum* and *U. lactuca*), 4 red seaweed (*C. crispus*, *G. gracilis*, *P. palmata*, and *P. dioica*), and 4 brown seaweed species (*E. bicyclis*, *F. vesiculosus*, *H. elongata*, and *U. pinnatifida*) were extracted with water at 20 °C for 10 or 20 min	ABTS varied between 51 (20 min) and 58% (10 min) when UAE was applied to *E. bicyclis*, thus exceeding optimized SLE, but not SWE	[85]
	4 brown seaweed species (*Sargassum aquifolium*, *S. cristaefolium*, *Sargassum polycystum*, *T. ornata*) were subjected to UAE at room temperature for 30 min (repeated three times) using a 1:10 (*w*/*v*) biomass/ethanol ratio and 30 kHz frequency	Extraction yield and total flavonoid contents were 5–8% and 400–700 mg quercetin/g dw, respectively, thus surpassing DR/SLE yields	[90]
	11 brown seaweed species (*A. esculenta*, *A. nodosum*, *F. serratus*, *F. spiralis*, *F. vesiculosus*, *Halidrys siliquosa*, *H. elongata*, *L. digitata*, *L. hyperborea*, *L. saccharina*, and *Pelvetia caniculata*) were extracted with ethanol/water (3:7, 5:5 or 7:3, *v*/*v*) using a biomass/solvent ratio of 1:10, *w*/*v*, at room temperature for 10 or 30 min and subjected to either 35 or 130 kHz	The optimal conditions for *F. vesiculosus* were applied to all other species: 5:5, *v*/*v*, ethanol/water for 30 min and using 35 kHz; extraction yield varied between 20.4% in *F. serratus* and 36.9% in *L. hyperborea*, being UAE yield improved by 1.5–2.2 fold in comparison to SLE in all tested seaweeds	[92]
	2 red seaweed species (*E. denticulatum* and *K. alvarezii*) and 2 brown seaweed species (*S. binderi* and *T. ornata*) were extracted by UAE and a parameter optimization was carried out: temperature (50, 70, 90 °C), pH (8–12), biomass/water ratio (1:33, 1:50, 1:100, *w*/*v*), and ultrasound power (75, 120, 150 W)	Polysaccharide extraction yield of 55% of the initial dry biomass weight was achieved in 15–30 min, while only 27% yield was attained after 2 h processing in a conventional extraction	[246]
WR/EAE	Brown seaweed *S. muticum* was extracted with 0.1 M phosphate buffer or 0.1 M acetate buffer at 40–60 °C using a 1:50 (*w*/*v*) biomass/solvent ratio and several alternative enzymes (Alcalase, Amylase, Celluclast, Protamex, Rapidase, Viscozyme)	In the concentration of 2–5% enzyme, solubilization yield was ~50% of the initial dry material, and also maximal phenolic content and antioxidant activity were achieved; Celluclast led to the highest solubilization yields	[247]
WR/PEF	Green seaweed *Ulva* sp. was extracted by PEF using water as solvent, variable voltage (20, 35, 50 kV), and variable number of 4–6 μs pulses (10, 20, 30, 40, 50) at 0.5 Hz frequency; a control extract with no voltage was also prepared	Compared to control, protein extraction yield increased by a factor of ~7 with PEF’s following conditions: 50 pulses of 50 kV, 247 kJ/kg fresh seaweed, and 70.3 mm electrode gap	[248]
WR/pHE	Red seaweed *Gracilaria* sp. was extracted by pHE in combination with PEF (3 Hz frequency, 200 pulses, 50 μs duration of the pulses, 1 kV), testing alternative sequential pH treatments, from 12 to 1 and from 1 to 12; PEF without pHE, pHE without PEF, and water-mediated extraction were also performed	Total solute release of ~10% with a combination of PEF and pHE, which did not improve with respect to pHE, but in comparison to PEF (~7%) and water-mediated extraction (~7%); however, protein yield was improved with respect to pHE, PEF, and water-mediated extraction, ~25% vs. ~15%, ~8%, and ~8%, respectively	[249]
	Brown seaweed *S. latissima* protein was extracted by pHE, being pH adjusted in a 2–13 range and optimization performed: temperature (4, 20, 50 °C), ratio between biomass and volume of water in the osmoshock step and alkaline extraction (1:20, 1:40, 1:60, *w*/*v*), osmotic shock duration (0, 1, 2, 16 h)	Maximum protein extraction, 34% of total protein, was reached at pH 12 and with 1:20, *w*/*v*, overall biomass/solvent; after a pH-shift combining alkaline extraction and acid precipitation, ~16% of seaweed protein recovery was achieved; osmoshocking significantly increased the yield	[250]
WR/UAE	Brown seaweed *S. muticum* was extracted with 0.1 M phosphate buffer or 0.1 M acetate buffer at 40–60 °C using a 1:50 (*w*/*v*) biomass/solvent ratio and 24 kHz frequency	Extraction yield varied between 21 and 40% of the initial dry material	[247]
	Brown seaweed *S. muticum* was extracted with water at 25 °C for 5, 10, 15, 20, 25, or 30 min using a 1:20 (*w*/*v*) biomass/solvent ratio, 40 kHz frequency, and 150 W power	Fucoidan extraction by UAE for 15 min resulted in a maximal sulfate content of 39.5 mg/g extract	[251]

## Data Availability

The data supporting the conclusions of this article will be made available by the authors on request.

## Abbreviations

The following abbreviations are used in this manuscript:

ABTS	2,2′-Azino-Bis(3-ethylbenzoThiazoline-6-Sulfonic acid
AlE	Alkali Extraction
APCI	Antioxidant Potency Composite Index
BaM	Ball Milling
BBD	Box–Behnken Design
BeM	Bead Milling
BMIA	1-Butyl-3-MethylImidazolium Acetate
BMIC	1-Butyl-3-MethylImidazolium Chloride
BMIDP	1-Butyl-3-MethylImidazolium Dibutyl Phosphate
BMITFB	1-Butyl-3-MethylImidazolium TetraFluoroBorate
ChC	Choline Chloride
CPAE	Cold Plasma-Assisted Extraction
DES	Deep Eutectic Solvent
DPPH	2,2-DiPhenyl-1-PicrylHydrazyl
DR	Dry Route
DW	Dry Weight
EAE	Enzyme-Assisted Extraction
EC_50_	Half Maximal Effective Concentration
EMIDP	1-Ethyl-3-Methyl-Imidazolium Dibutyl Phosphate
FRAP	Ferric Reducing Antioxidant Power
GAE	Gallic Acid Equivalent
HaM	Hammer Milling
HDC	HydroDynamic Cavitation
HHP	High Hydrostatic Pressure
HMF	5-hydroxyMethylFurfural
HPCD	High-Pressure Cell Disruption
HSH	High Speed Homogenization
ICPD/DIC	Instant Controlled Pressure Drop/‘Détente Instantanée Contrôlée’
IL	Ionic Liquid
IMTA	Integrated Multi-Trophic Aquaculture
LA	Lactic Acid
LCA	Life Cycle Analysis
MAE	Microwave-Assisted Extraction
NADES	Natural Deep Eutectic Solvent
OL	Osmotic Lysis
ORAC	Oxygen Radical Absorbance Capacity
PE	Percolation
PEF	Pulsed Electric Field
PGE	PhloroGlucinol Equivalent
PHB	PolyHydroxyButyrate
pHE	pH-shift Extraction
PLE	Pressurized Liquid Extraction
RE	Reflux Extraction
RSM	Response Surface Methodology
SFE	Supercritical Fluid Extraction
SLE	Solid–Liquid Extraction
SP	Screw Pressing
SPE	Solid-Phase Extraction
SPME	Solid-Phase MicroExtraction
SWE	Sub-critical Water Extraction
TBTDPC	TriButylTetraDecylPhosphonium Chloride
TMAH	TetraMethylAmmonium Hydroxide
UAE	Ultrasound-Assisted Extraction
WR	Wet Route
